# Taxonomic note of *Parnassia* (Celastraceae) in China: a reassessment of Subsect. Xiphosandra

**DOI:** 10.3897/phytokeys.114.30551

**Published:** 2018-12-20

**Authors:** Huiying Yu, Feiyi Guo, Yumin Shu, Zhixiang Zhang

**Affiliations:** 1 Laboratory of Systematic Evolution and Biogeography of Woody Plants, College of Nature Conservation, Beijing Forestry University, Beijing 100083, China; 2 Institute of Plant Adaptation and Utilization in Southwest Mountain, College of Life Science, China West Normal University, Nanchong, Sichuan 637002, China; 3 Museum of Beijing Forestry University, Beijing 100083, China

**Keywords:** Subsect. *Xiphosandra*, Celastraceae, new synonym, taxonomy

## Abstract

*P.brevistyla*, *P.delavayi* and *P.leptophylla*. belong to Celastraceae, ParnassiaL.,Sect.NectarotrilobosDrude,Subsect.Xiphosandra (Franch.) Ku. Due to lack of material, the reliability of their taxonomic characteristics remained unknown and all three species have been retained. Following extensive field investigations, population sampling and examination of specimens, we determined that the previously used characteristics to distinguish these three species, i.e. the shape of basal leaves and the depth of staminode lobes, vary continuously within populations and should not be used to distinguish separate species. Therefore, *P.brevistyla* and *P.leptophylla* are hereby designated as synonyms of *P.delavayi*.

## Introduction

*Parnassia* L. (Linnaeus 1753) is a genus that is predominantly distributed in the arctic and temperate zones of the Northern Hemisphere; it is most speciose in China and the Himalayas (Simmons 2004). All species of *Parnassia* are perennial herbs, glabrous; flowers solitary, borne on unbranched scape, actinomorphic, hermaphrodite; staminodes 5, inserted opposite to petals, terete and entire or flat and divided into lobes or filiform rays. The genus was traditionally placed in the family Saxifragaceae (Hooker and Thompson 1858, Engler 1930, Cronquist 1981, Ku 1995, Ku and Hultgård 2001); however, it was recently transferred to Celastraceae (APG IV 2016, Ball 2016) based on molecular evidence (Soltis et al. 1997, Soltis et al. 2000, Zhang and Simmons 2006, APG IV 2016). The latest updated checklist of the genus recognised 61 species, 2 subspecies, 11 varieties and 1 forma (Shu et al. 2017).

**Figure 1.**
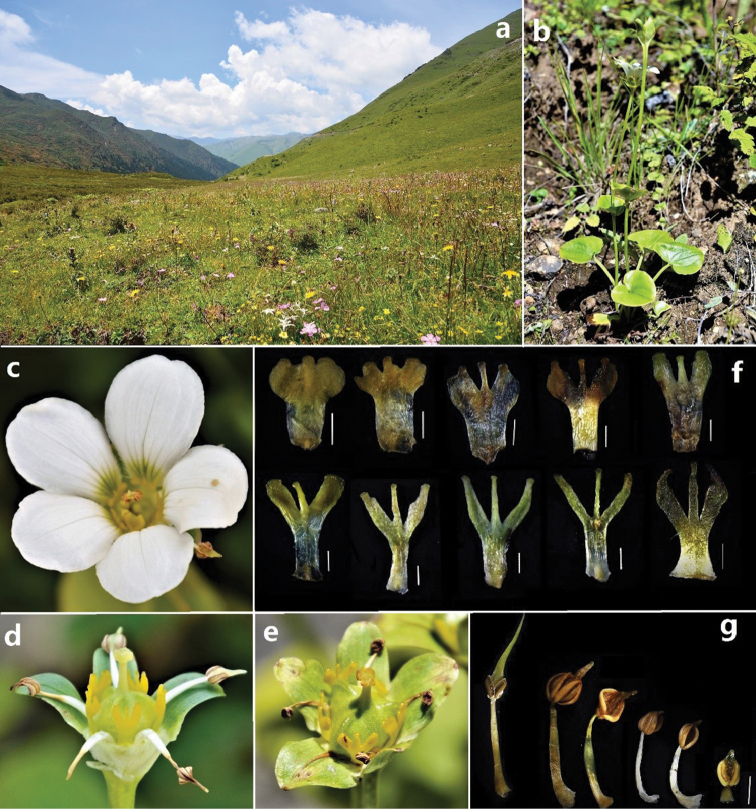
*Parnassiadelavayi*: habitat (**a**), individual (**b**), flower (**c**), fruits (**d, e**), staminodes (**f**) and stamens (**g**). Scale bar: 1 mm.

Subsect. Xiphosandra (Franch.) Ku (1987) has been recognised as consisting of those species in which the stamen connectives apically project as lanceolate appendages (Fig. 1g). It includes three species: *P.brevistyla*, *P.delavayi* and *P.leptophylla*. Based on unique morphological characteristics (i.e. the stamen connectives apically project as lanceolate appendages), the three species were placed in Sect. Xiphosandra Franch. (Franchet 1897) with the support of Wu et al. (2003). Alternatively, most researchers placed them into Subsect. Xiphosandra of Sect. Nectarotrilobos Drude (Ku 1987, Wu 2005) or directly into Sect. Nectarotrilobos due to their flat and 3-lobed staminodes (Nekrassova 1927, Engler 1930, Handel-Mazzetti 1941).

**Figure 2.**
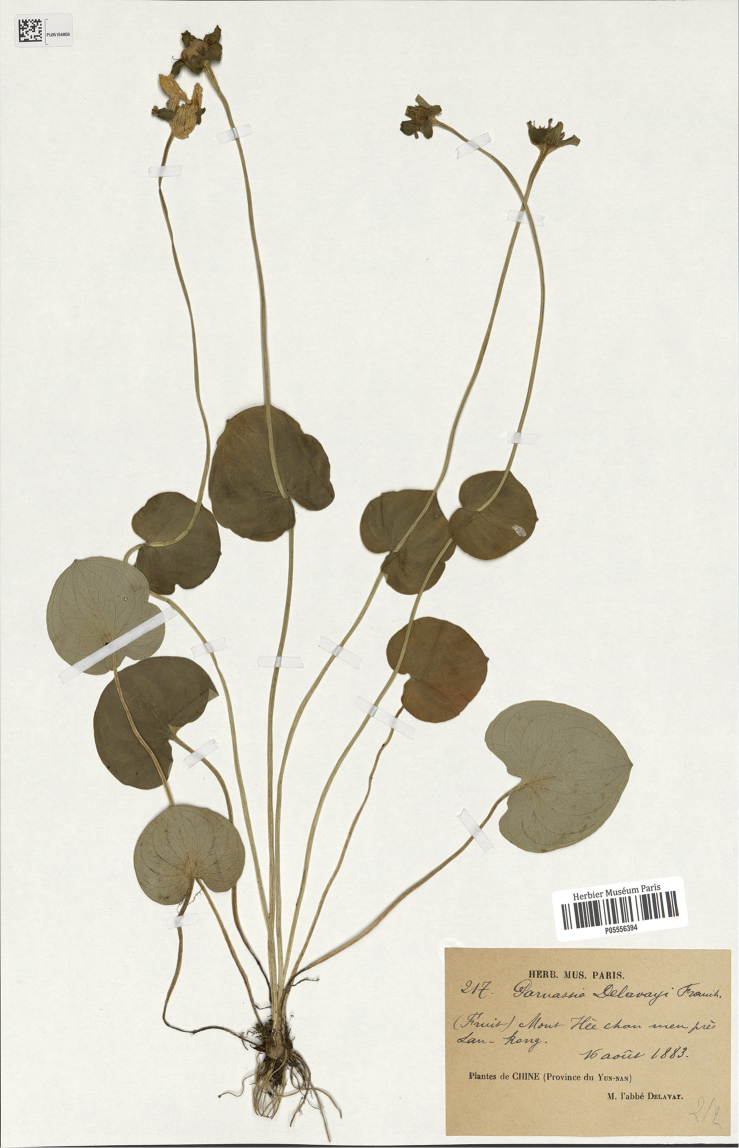
*Parnassiadelavayi*, lectotype (P05556394).

**Figure 3.**
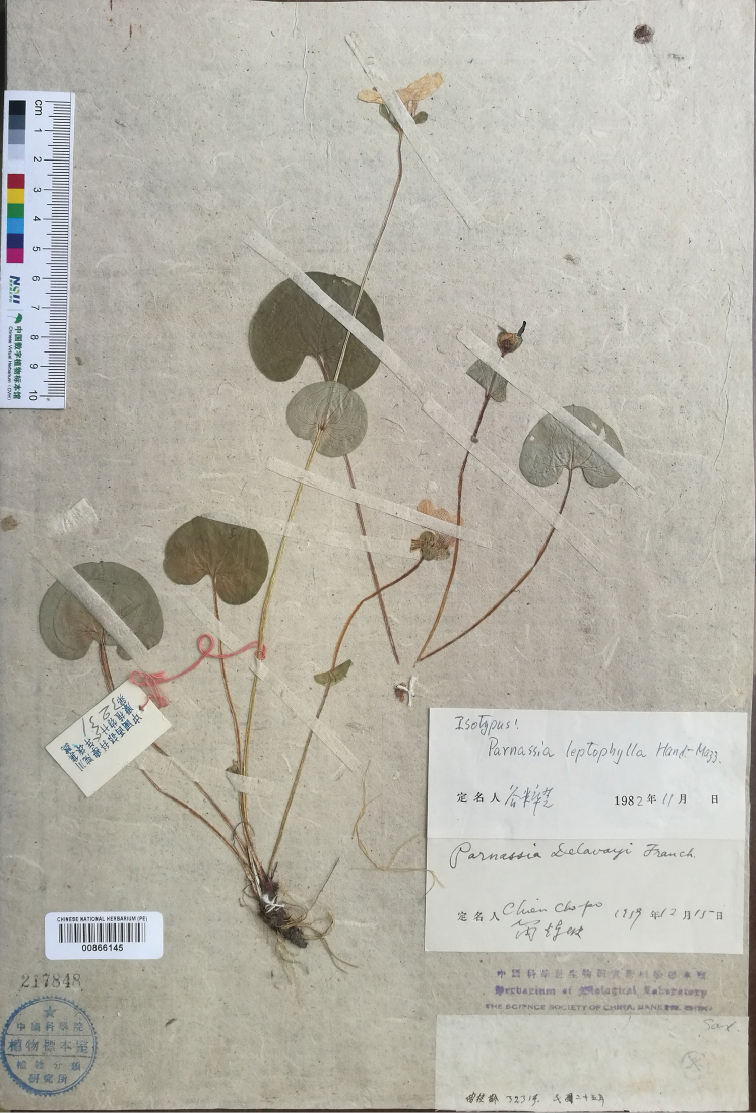
*Parnassialeptophylla*, lectotype (PE00866146).

*Parnassiadelavayi* Franch. was published based on three collections (*Delavay 217*, *Delavay s.n.* and *Delavay Parnassia n. 2*) (Franchet 1896). Then, ParnassiadelavayiFranch.var.brevistyla Brieger was proposed in 1922 based on specimens collected from Tibet, China, 3750 m and Schenhsi, China, 2600 m, *s.n*. (Brieger 1922). Handel-Mazzetti (1931) promoted Parnassiadelavayivar.brevistyla into the new species *Parnassiabrevistyla* (Brieg.) Hand.-Mazz. Handel-Mazzetti (1941) published 10 new taxa, including *Parnassialeptophylla* Hand.-Mazz., on the basis of a specimen collected from Baoxing, Sichuan, China—*KL Chu 3231*. Since the authors did not specify the holotype of *Parnassiadelavayi* or *P.leptophylla* in their publications, P00709352 (Fig. 2) was designated as the lectotype of *P.delavayi* and PE00866146 (Fig. 3) was designated as the lectotype of *P.leptophylla* by Shu et al. (2017). However, according to the information provided by the original description of *P.brevistyla*, these types of *P.brevistyla* were not found after reviewing the specimens and high-resolution images of specimens in major herbaria, which was also mentioned by Stafleu and Mennega (1995).

Based on the descriptions of *P.brevistyla*, *P.delavayi* and *P.leptophylla*, the differences amongst them mainly consist of two characteristics: the shape of leaf blades and the depth of staminode lobes (Ku 1995, Table 1). With regard to the shape of blades: *P.brevistyla* has ovate-cordate basal leaves, while the other two species have reniform or occasionally suborbicular basal leaves. With regard to the depth of staminode lobes: *P.brevistyla* staminodes are shallowly 3-lobed and the length of lobes is 1/6 or less of staminodes’ length; *P.delavayi* staminodes are shallowly 3-lobed or to half their length; *P.leptophylla* staminodes are 3-lobed for ca. 3/4 of their length.

Through observation of herbarium specimens, we found that the three species of Subsect. Xiphosandra are extremely similar in morphology and there is obvious overlap amongst *P.brevistyla*, *P.delavayi* and *P.leptophylla* in the shape of leaf blades and the depth of staminode lobes. Moreover, during extensive field work, we found that the shape of basal leaves and the depth of staminode lobes varied significantly within populations, even sometimes within individuals. Therefore, the aim of this study was to clarify the classification relationship amongst *P.brevistyla*, *P.delavayi* and *P.leptophylla* by re-evaluating their morphological characteristics, particularly the shape of blades and the depth of staminode lobes.

**Table 1.** Morphological characters of the specimens amongst *Parnassiabrevistyla*, *P.delavayi* and *P.leptophylla*.

	*** P. brevistyla ***	*** P. delavayi ***	*** P. leptophylla ***
Leaves	Ovate-cordate	Reniform, occasionally suborbicular	Reniform, occasionally suborbicular
Staminodes	Shallowly 3-lobed,the length of lobes is 1/6 or less of their length	Shallowly 3-lobed or to half their length	3-lobed for ca. 3/4 of their length

## Material and methods

Specimens from **BJFC**, **CDBI**, **IMC**, **KUN**, **QFNU**, **P**, **PE**, **SM**, **SZ** and **WCNU** (Suppl. material 1) were examined during this study. High-resolution photographs were also captured. The measurements provided herein were mostly obtained from dried herbarium specimens. Since the shape of both the leaf base and the apex are basically identical, the leaf shape is defined by the aspect ratio of leaves in this study. The primary examined populations were in Chongqing, Gansu, Guizhou, Hebei, Henan, Hubei, Hunan, Shanxi, Sichuan, Tibet and Yunnan. These populations include the type localities of *P.brevistyla*, *P.delavayi* and *P.leptophylla* (Taibai, Shanxi; Baoxing, Sichuan; Heqing, Dali). To ensure the data are fully comprehensive, we have recorded all measurable traits, such as sepals and petals, in each population.

An Olympus SZX16 dissecting microscope was used for observations and an Olympus DP72 cooled digital colour camera was used to photograph leaves and staminodes. TpsDig software: Version 2.17 (http://life.bio.sunysb.edu/ee/rohlf/software.html) was used to measure the lengths and widths of leaves, thus improving the accuracy of experimental results.

**Figure 4.**
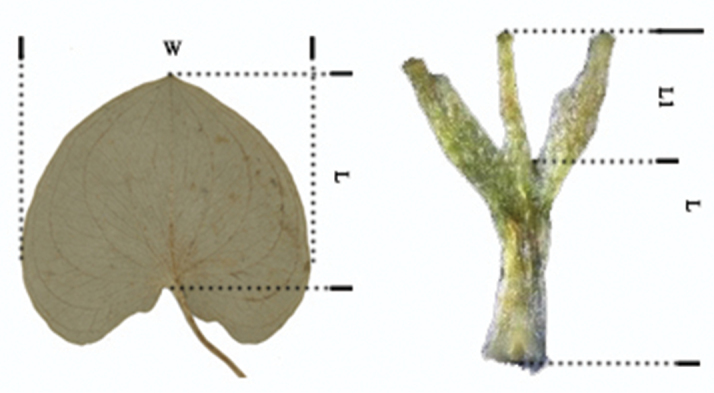
Measurements of leaves and staminodes: W: the width of leaves; L: the length of leaves / staminodes; L1: the length of central lobes.

Overlapping or non-overlapping standard deviations can be used to determine if there is continuous or discontinuous variation of a quantitative trait between two or more groups, thus providing evidence for species differentiation or merging. This approach was applied to the aspect ratio of leaves (the width of leaves / the length of leaves) and the depth of staminode lobes (the length of central lobes / the length of staminodes) (Fig. 4). A total of 100 populations from 11 provinces were studied. To ensure the universality and comprehensive nature of the data, we measured all available individuals.

## Results

**Figure 5.**
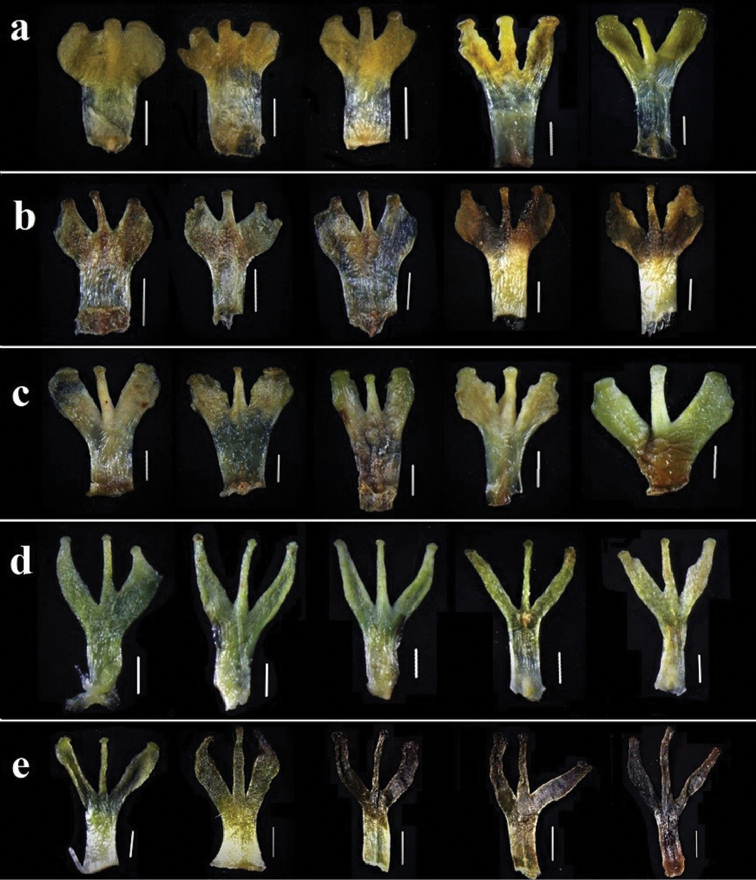
Variation of staminodes: **a, b** represent variation of the depth of staminode lobes in population of Kangding, Sichuan **c** represents variation of the depth of staminode lobes in population of Baoxing, Sichuan (the type locality of *Parnassialeptophylla*) **d** represents variation of the depth of staminode lobes in population of Cangshan, Dali (the type locality of *P.delavayi*) **e** represents variation of the depth of staminode lobes in population of Mabian, Sichuan. Scale bar: 1 mm.

**Figure 6.**
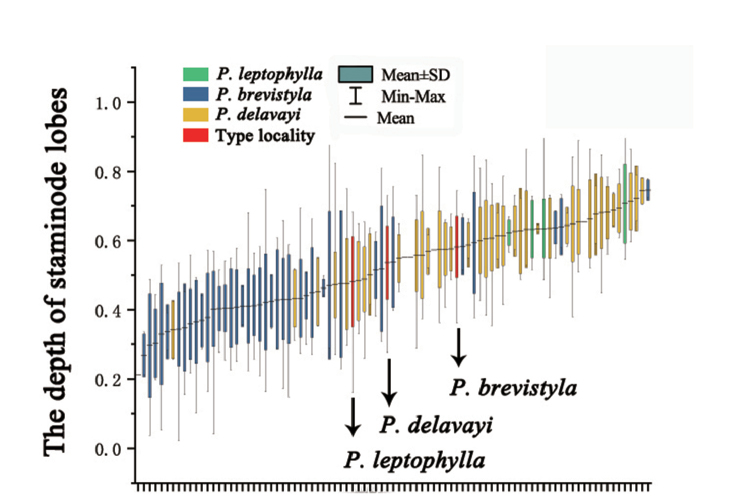
The standard deviation analysis of the depth of staminode lobes.

The morphological observations showed that the depth of staminode lobes varies considerably in the sampled populations, ranging from shallowly 3-lobed to 3-lobed for ca. 2/3 of their length (Fig. 5). Furthermore, the standard deviation analysis of the depth of staminode lobes also showed statistical continuity amongst populations of *P.brevistyla*, *P.delavayi* and *P.leptophylla*. As shown in Fig. 6, the variation of the depth of staminodes in the specimens collected from Baoji County, Heqing County and Baoxing County, where the types of *P.brevistyla*, *P.delavayi* and *P.leptophylla* were collected, is within the variation range of the population of *P.brevistyla*. There is also significant overlap of the depth of staminode lobes amongst the three species. Thus, *P.brevistyla*, *P.delavayi* and *P.leptophylla* show no differences in the depth of staminode lobes.

**Figure 7.**
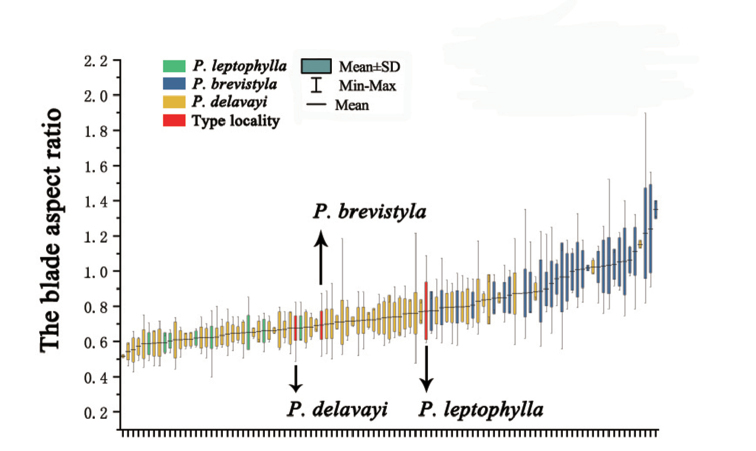
The standard deviation analysis of the blade aspect ratio.

The shape of basal leaves has also been considered to be a distinguishing characteristic in previous treatments (Ku 1987, 1995). However, we found that the shape of basal leaves varies greatly within populations. Based on field experience, we found that their leaf shape varies dramatically, not only amongst different regions, but also in the same population. Shapes range from reniform, broadly cordate, to cordate and ovate-cordate. As shown in Fig. 7, the variation of the blade aspect ratio in the specimens collected from type localities of *P.brevistyla*, *P.delavayi* and *P.leptophylla* is within the variation range of *P.brevistyla* populations. Furthermore, a significant overlap was found for the blade aspect ratio amongst these three species with respect to the observed ranges as well as the standard deviations (Fig. 7). Based on this continuous variation, it is difficult to find a dividing line to distinguish different leaf shapes.

In summary, *P.brevistyla*, *P.delavayi* and *P.leptophylla* show no differences in the shape of blades and the depth of staminode lobes. Thus, we treated *P.brevistyla* and *P.leptophylla* as new synonyms of *P.delavayi*.

## Discussion

Hong (2016) proposed a new operational species concept called the morphological-biological species concept which emphasises that two or more qualitative traits or statistically discontinuous quantitative characters should exist between two species (Liu 2016). We applied this species concept in this study.

Taxonomically delimited species can be regarded as an assemblage of populations or individuals that have common morphological features and show distinct morphological discontinuity with other such assemblages in a number of characteristics (Singh 2004). That is, individuals of a given species show continuous variation in some characteristics. However, due to limitations of herbarium material, researchers can only observe a certain state of an individual within a population, rather than its variation within populations. Therefore, field investigation and random sampling within populations are important components of taxonomic studies (Xu et al. 2017). In this study, through both random sampling and statistical analysis within populations, the variability of morphological characteristics used in previous taxonomic treatments were analysed and reconsidered to avoid taxonomic artifacts that may be caused by undersampling.

We determined that both major characteristics that were previously used to distinguish *P.brevistyla*, *P.delavayi* and *P.leptophylla*, i.e. the shape of blades and the depth of staminode lobes, vary remarkably and continuously. Therefore, these characteristics should not be used to justify the recognition of three species. Furthermore, based on our examination of herbarium specimens as well as field observations, we found no other distinct characteristics to separate *P.brevistyla*, *P.delavayi* and *P.leptophylla* at the species level.

Furthermore, the distributions of these three species are continuous and overlap. Therefore, since these three species lack clear morphologic distinction and overlap in their distribution range, we hereby reduce *P.brevistyla* and *P.leptophylla* to synonyms of *P.delavayi*.

## Taxonomic treatment

### Parnassia delavayi

Taxon classificationPlantaeCelastralesCelastraceae

Franchet, 1896: 267

Fig. 2

=Parnassiabrevistyla (Brieg.) Hand.-Mazz., 1931: 434. Syn. nov.
≡ParnassiadelavayiFranch.var.brevistyla Brieger, 1922: 400. Syn. nov. Type: China. Tibet, ‘*Beju-Batang, Nadelwalder bei Chieda am Anstieg zum Passe Mala*’, 3500 m alt. (syntypes: not located); China. Schenhsi, Qingling, Taibaishan, Tempels Wan-schuen-gou, 2600 m alt., *s.n. 2720* (syntypes: not located).
=Parnassialeptophylla Hand.-Mazz., 1941: 120. Syn. nov. (Fig. 3). Type: China. Sichuan, Baoxing, *KL Chu 3231* (lectotype designated by Shu et al. 2017: PE00866146!; isolectotypes: IBSC0145403!, PE00865960!, SZ00179815!). Fig. 3.

#### Type.

CHINA. Yunnan, Eryuan (Lan-kong), Hee Chanmen, 2800 m alt., 16 August 1883, *Delavay 217* (lectotype designated by Shu et al. 2017: P05556394!; isolectotypes: P00709355!, P00709356!, P05556395!, P06392624!); CHINA. Yunnan, Eryuan, Lanho, Yanginchan, 7 August 1883, *Delavay 130* (syntypes: P00709357!, P00709358!, P06392623!)

#### Description.

Stems 1–5, 10–40 cm, with 1 cauline leaf proximally or near middle. Basal leaves 3–7(10); petiole 2–16 cm; blade reniform, cordate or ovate-cordate, 1–4 × 1–4.5 cm, base deeply cordate to subcordate, apex rounded, apiculate. Flower 2–4.5 cm in diam.; hypanthium turbinate or campanulate. Sepals oblong, ovate to obovate, 4–13 × 2–8 mm, margin entire, apex rounded-obtuse. Petals white, sometimes green at base, obovate or oblong-obovate, 8–25 × 5–12 mm, base attenuate, margin erose in proximal 1/3, apex rounded or acute. Anthers ellipsoid, connective projected at apex into a lanceolate appendage, to 5 mm; filaments ca. 5.5 mm; staminodes 3–6 mm, shallowly 3-lobed or to half their length, rarely lateral ones 2-lobulate, lateral lobes usually wider than central one. Ovary superior, subglobose; stigma 3-lobed. Capsule obovoid with 3 thickened, longitudinal angles. Seeds brown, glossy, oblong. 2n = 14.

## Supplementary Material

XML Treatment for Parnassia delavayi

## Supplementary materials

Supplementary material 1 A list of herbarium specimens examined Data type: species data This is an open access article distributed under the terms of the Creative Commons Attribution License (CC BY 4.0), which permits unrestricted use, distribution, and reproduction in any medium, provided the original author and source are credited.Huiying Yu, Feiyi Guo, Yumin Shu, Zhixiang Zhang
