# Supplementary material for: Taxonomic note of Parnassia (Celastraceae) in China: a reassessment of Subsect. Xiphosandra
Source: PhytoKeys. 2018 Dec 20;(114):43–54. doi: 10.3897/phytokeys.114.30551 (PMC6308226; doi:10.3897/phytokeys.114.30551)
Supplement: Supplementary material 1 — A list of herbarium specimens examined [file phytokeys-114-043-s001.pdf]

APPENDIX 1. **A list of herbarium specimens examined.**

Collector & Collecting numbers	Herbaria	Bar code	Collecting date
Parnassia brevistyla			
Bing-Zhi Ni et al. 197	CDBI	CDBI0037134	19640704
Bing-Zhi Ni et al. 197	CDBI	CDBI0037135	19640704
Bing-Zhi Ni et al. 326	CDBI	CDBI0037133	19640808
Bing-Zhi Ni et al. 326	CDBI	CDBI0039060	19640808
Bing-Zhi Ni et al. 9010	CDBI	CDBI0038505	19750625
Bing-Zhi Ni et al. 9010	CDBI	CDBI0038506	19750625
Dao-Fu HuiCun et al. 739	CDBI	CDBI0040209	19900704
Dao-Fu HuiCun et al. 813	CDBI	CDBI0040207	19900706
Dao-Fu HuiCun et al. 813	CDBI	CDBI0040208	19900706
Qing-Sheng Zhao 6576	CDBI	CDBI0039157	19780813
Qing-Sheng Zhao 6576	CDBI	CDBI0039158	19780813
Qing-Sheng Zhao 6576	CDBI	CDBI0039159	19780813
SiChuan Vegetation Research Team 2167	CDBI	CDBI0037136	19730719
SiChuan Vegetation Research Team 2167	CDBI	CDBI0037137	19730719
SiChuan Vegetation Research Team 2167	CDBI	CDBI0037138	19730719
SiChuan Vegetation Research Team 2579	CDBI	CDBI0037139	19730820
SiChuan Vegetation Research Team 2579	CDBI	CDBI0037140	19730820
SiChuan Vegetation Research Team 2579	CDBI	CDBI0037141	19730820
SiChuan Vegetation Research Team 3151	CDBI	CDBI0037142	19730812
SiChuan Vegetation Research Team 3151	CDBI	CDBI0037146	19730812
SiChuan Vegetation Research Team 3151	CDBI	CDBI0037149	19730812
SiChuan Vegetation Research Team 4329	CDBI	CDBI0037143	19730922
SiChuan Vegetation Research Team 4329	CDBI	CDBI0037144	19730922
SiChuan Vegetation Research Team 4329	CDBI	CDBI0037145	19730922
Tai-Chang Wei 20612	CDBI	CDBI0037132	19790717
Tai-Chang Wei 20612	CDBI	CDBI0037154	19790717
Tibet Team 003828	CDBI	CDBI0039077	19810806
Tibet Team 003828	CDBI	CDBI0039978	19810806
Tibet Team 3957	CDBI	CDBI0039519	19810809

Tibet Team 4374	CDBI	CDBI0039914	19810827
Tibet Team 4628	CDBI	CDBI0039316	19810811
Tibet Team 4628	CDBI	CDBI0039898	19810811
Tibet Team 4754	CDBI	CDBI0039743	19810812
Tibet Team 4754	CDBI	CDBI0039883	19810812
Tibet Team 4758	CDBI	CDBI0039742	19810812
Unknown Collector 1057	CDBI	CDBI0039799	19940628
Unknown Collector 1222	CDBI	CDBI0037147	19720830
Unknown Collector 1222	CDBI	CDBI0037148	19720830
Unknown Collector 5777	CDBI	CDBI0038378	19740807
Unknown Collector 5777	CDBI	CDBI0039054	19740807
Unknown Collector 5777	CDBI	CDBI0039055	19740807
Unknown Collector 6004	CDBI	CDBI0038359	19580720
Unknown Collector 6004	CDBI	CDBI0038360	19580720
Unknown Collector 6421	CDBI	CDBI0039571	19580802
Unknown Collector 6421	CDBI	CDBI0039572	19580802
Unknown Collector 7325	CDBI	CDBI0038374	19740707
Unknown Collector 7325	CDBI	CDBI0038375	19740707
Unknown Collector 7325	CDBI	CDBI0038376	19740707
Unknown Collector 7388	CDBI	CDBI0039241	19740710
Unknown Collector 7388	CDBI	CDBI0039242	19740710
Unknown Collector 7391	CDBI	CDBI0038502	19740710
Unknown Collector 7391	CDBI	CDBI0039564	19740710
Unknown Collector 7391	CDBI	CDBI0039568	19740710
Unknown Collector 9335	CDBI	CDBI0038508	19750718
Xiao-Hong Hu 29337	CDBI	CDBI0037152	19820812
Xiao-Hong Hu 29337	CDBI	CDBI0037153	19820812
Yu-Lan Peng Gaoxf0302	CDBI	CDBI0205550	20080718
Yu-Lan Peng Gaoxf0302	CDBI	CDBI0205554	20080718
Yu-Lan Peng Gaoxf0342	CDBI	CDBI0205552	20080718
Yu-Lan Peng Gaoxf0342	CDBI	CDBI0205556	20080718
Ze-Sun Yu et al. 6451	CDBI	CDBI0037161	19740705
Ze-Sun Yu et al. 6451	CDBI	CDBI0039567	19740705
Ze-Sun Yu et al. 6451	CDBI	CDBI0039725	19740705
Ze-Sun Yu et al. 6451	CDBI	CDBI0039726	19740705
De-Jun Yu 6885	KUN	KUN0437011	19370707
Ding-Wu & Yi-Ping Wang 2011001	KUN	KUN	
Ding-Wu et al. 2014	KUN	KUN	20020914
Ding-Wu et al. 5078	KUN	KUN	20050817
Ding-Wu et al. 5090	KUN	KUN	20080818
Ding-Wu et al. 5120	KUN	KUN	20050819
En-De Liu et al. 7049	KUN	KUN1341578	20170706
Jing-Sheng Yang 7980	KUN	KUN0437446	19760718

Kai-Yong Lang et al. 1219	KUN	KUN0436995	19830819
Kai-Yong Lang et al. 1219	KUN	KUN0436995	19820819
Kai-Yong Lang et al. 2321	KUN	KUN0437014	19830724
Kai-Yong Lang et al. 2532	KUN	KUN0436993	19830731
Kai-Yong Lang et al. 2643	KUN	KUN0436996	19830802
Kai-Yong Lang et al. 2806	KUN	KUN0436994	19830807
SiChuan Vegetation Research Team 2167	KUN	KUN0437008	19730719
SiChuan Vegetation Research Team 2579	KUN	KUN0437007	19730820
SiChuan Vegetation Research Team 3151	KUN	KUN0437006	19730815
SiChuan Vegetation Research Team 4329	KUN	KUN0437005	19730922
Tibet Team 12215	KUN	KUN0437000	19830721
Tibet Team 12215	KUN	KUN0437001	19830721
Tibet Team 12295	KUN	KUN0437002	19830723
Tibet Team 12295	KUN	KUN0437003	19830723
Tibet Team 3957	KUN	KUN0436998	19810809
Tibet Team 3957	KUN	KUN0436999	19810809
Tibet Team 4375	KUN	KUN0436997	19810827
Tibet Team 4628	KUN	KUN0436988	19810811
Tibet Team 4628	KUN	KUN0436989	19810811
Tibet Team 4754	KUN	KUN0436990	19810812
Tibet Team 4754	KUN	KUN0436991	19810812
Tibet Team 4758	KUN	KUN0436992	19810812
Tibet Team 751933	KUN	KUN0437015	19750910
Tibet Team 751933	KUN	KUN0437016	19750910
Unknown Collector 1535	KUN	KUN0437009	19590703
Unknown Collector 4528	KUN	KUN0437004	19600807
Wen-Bin Yu et al. 5051	KUN	KUN	20060706
You-Wen Pan 92013	KUN	KUN0436987	19920705
Ze-Yun Zhang & Hong-Fu Zhou 22893	KUN	KUN0437010	19570717
Zhi-Ping Huang 1481	KUN	KUN1205223	19300718
Zhi-Ping Huang 1481	KUN	KUN1205226	19300718
ZhongDian Team 3666	KUN	KUN0437444	19630721
ZhongDian Team 3666	KUN	KUN0437445	19630721
ZhongDian Team 906	KUN	KUN0437447	19620816
ZhongDian Team 906	KUN	KUN0437448	19620816
Zhu et.al 1996	KUN	KUN0491230	19990910
Boufford DE et al 28473	P	P05494760	19980709
Boufford DE et al 28738	P	P05494758	19980716
Soulie	P	P03331597	19030000

Soulie 304	P	P05608127	18930000
ChuanXi Team 311	PE	PE00865760	19630724
ChuanXi Team 311	PE	PE00865761	19630724
ChuanXi Team 311	PE	PE00865762	19630724
CS Liu 1095	PE	PE00865796	19340816
CS Liu 1095	PE	PE00865800	19340816
Da-Hai Zhu et al. 4649	PE	PE	20070811
feng 1965	PE	PE01982426	19390810
FT Wang 21440	PE	PE00865797	19300621
Hai-Ning Qin et al. 103	PE	PE	20010827
HengDuanShan Vegetation Team 4977	PE	PE	19830812
HengDuanShan Vegetation Team 4977	PE	PE	19830812
HengDuanShan Vegetation Team 4977	PE	PE	19830812
HengDuanShan Vegetation Team 4977	PE	PE	19830812
JF Rock 12790	PE	PE00865755	19250700
Jun-Sheng Ying & De-Yuan Hong 650807	PE	PE00865816	19650730
Jun-Sheng Ying & De-Yuan Hong 650807	PE	PE00865817	19650730
Jun-Sheng Ying & De-Yuan Hong 650807	PE	PE00865818	19650730
Kai-Yong Lang et al. 1219	PE	PE00864200	19820819
Kai-Yong Lang et al. 1219	PE	PE01982471	19820819
Kai-Yong Lang et al. 2321	PE	PE00864199	19830724
Kai-Yong Lang et al. 2321	PE	PE01982479	19830724
Kai-Yong Lang et al. 2532	PE	PE00864187	19830731
Kai-Yong Lang et al. 2532	PE	PE00864188	19830731
Kai-Yong Lang et al. 2643	PE	PE00861969	19830802
Kai-Yong Lang et al. 2643	PE	PE00861970	19830802
Kai-Yong Lang et al. 2806	PE	PE00864193	19830807
Kai-Yong Lang et al. 2806	PE	PE00864194	19830807
Kai-Yong Lang et al. 680	PE	PE00865763	19820727
Kai-Yong Lang et al. 680	PE	PE00865764	19820727
Kai-Yong Lang et al. 680	PE	PE01982420	19820727
KangZang Plant Research Team 10-0190	PE	PE01873304	20100719
KangZang Plant Research Team 10-0281	PE	PE01873299	20100720
KangZang Plant Research Team 10-0501	PE	PE01873298	20100723

KangZang Plant Research Team 10-2133	PE	PE01873300	20100817
Ke-Jian Guan et al. 221	PE	PE00865757	19630723
Ke-Jian Guan et al. 221	PE	PE00865758	19630723
Ke-Jian Guan et al. 221	PE	PE00865759	19630723
KL Chu 7689	PE	PE00865802	19400812
Li-Min Lu 2008338	PE	PE	20080719
Li-Min Lu 2008338	PE	PE	20080719
Li-Song Wang & Jian-Fei Ye 130	PE	PE	20090807
Li-Song Wang & Jian-Fei Ye 130	PE	PE	20090807
NanShuiBeiDiao Team 4528	PE	PE01982469	19600807
NanShuiBeiDiao Team 9550	PE	PE00865770	19610722
NanShuiBeiDiao Team 9550	PE	PE00865772	19610722
Pu-Xiong Li 10456	PE	PE00865771	19580808
Sheng-Xiang Yu et al. 5085	PE	PE	20110808
Sheng-Xiang Yu et al. 5085	PE	PE	20110808
Sheng-Xiang Yu et al. 5106	PE	PE	20110808
Sheng-Xiang Yu et al. 5106	PE	PE	20110808
Sheng-Xiang Yu et al. 5106	PE	PE	20110808
Sheng-Xiang Yu et al. 5106	PE	PE	20110808
SiChuan Vegetation Research Team 2579	PE	PE00861975	19730820
SiChuan Vegetation Research Team 3151	PE	PE00861976	19730815
SiChuan Vegetation Research Team 2167	PE	PE00861977	19730719
SiChuan Vegetation Research Team 4329	PE	PE00861974	19730922
Su Jiang & Cun-Li Jin 01535	PE	PE00865767	19590703
Su Jiang & Cun-Li Jin 02273	PE	PE00865766	19590722
Su Jiang & Cun-Li Jin 02659	PE	PE00865765	19590801
Tibet Plant Team 9539	PE	PE00865821	19760821
Tibet Plant Team 9539	PE	PE00865822	19760821
Tibet Plant Team 9565	PE	PE00865820	19760821
Tibet Plant Team 9634	PE	PE00865819	19760820
Tibet Team 12295	PE	PE00861971	19830723
Tibet Team 12295	PE	PE01982472	19830723
Tibet Team 3828	PE	PE00861973	19810806
Tibet Team 3828	PE	PE00865769	19810806
Tibet Team 3957	PE	PE00864198	19810809
Tibet Team 3957	PE	PE01982478	19810809
Tibet Team 4375	PE	PE00864195	19810827
Tibet Team 4376	PE	PE00864196	19810827
Tibet Team 4628	PE	PE00864191	19810811

Tibet Team 4628	PE	PE00864192	19810811
Tibet Team 4754	PE	PE00864189	19810812
Tibet Team 4754	PE	PE00864190	19810812
Tibet Team 4758	PE	PE00864197	19810812
TP Wang 5580	PE	PE00865753	19360804
TP Wang 5580	PE	PE00865752	19360804
TP Wang 5580	PE	PE00865754	19360804
TT Yu 12232	PE	PE00865806	
TT Yu 12232	PE	PE00865811	19370717
TT Yu 12952	PE	PE00865812	19370828
TT Yu 12952	PE	PE00865813	19370828
TT Yu 12952	PE	PE00865815	
TT Yu 6885	PE	PE00865810	19370706
TT Yu 6885	PE	PE00865814	
TT Yu 2301	PE	PE00865794	19330713
Unknown Collector 10105	PE	PE00865780	19750629
Unknown Collector 10180	PE	PE00865781	19750708
Unknown Collector 32000	PE	PE00861980	19580606
Unknown Collector 5777	PE	PE00865779	19740807
Unknown Collector 7325	PE	PE00865774	19740707
Unknown Collector 7391	PE	PE00865773	19740710
Unknown Collector 7900	PE	PE00862147	19840917
Unknown Collector 9010	PE	PE00865777	19750625
Unknown Collector 9335	PE	PE00865778	19750718
Unknown Collector s.n	PE	PE00865808	
Wei-Lie Chen et al. 7900	PE	PE	19840717
Wei-Lie Chen et al. 7900	PE	PE	19840717
Wei-Lie Chen et al. 7900	PE	PE	19840717
Wei-Lie Chen et al. 7900	PE	PE	19840717
Wei-Lie Chen et al. 8671	PE	PE00862146	19840728
Wen-Cai Wang s. n.	PE	PE00861972	19860914
Wen-Guang Hu & Zhu He 10922	PE	PE00865792	19510818
Wen-Guang Hu & Zhu He 10972	PE	PE00865789	19510820
Wen-Guang Hu & Zhu He 11130	PE	PE01982467	19510906
Wen-Pei Fang 3549	PE	PE00865798	19280925
Wen-Pei Fang 4303	PE	PE00865795	19280821
Wen-Pei Fang 4303	PE	PE00865799	19280821
Xian-Jin Yang 3686	PE	PE00865805	
Xin Li & Ji-Xi Zhou 73917	PE	PE00865788	19570731
Xin Li 71548	PE	PE00865786	19570618
Xin Li 73027	PE	PE00865785	19570708
Xin Li 77795	PE	PE00865783	19580618
Xing-Ling Jiang 36411	PE	PE00865929	19530723

Xiu-Shi Zhang & You-Xian Ren 5941	PE	PE00865784	19580717
Xiu-Shi Zhang & You-Xian Ren 6004	PE	PE00861979	19580720
Xiu-Shi Zhang & You-Xian Ren 6348(6350)	PE	PE00861978	19580728
Xiu-Shi Zhang & You-Xian Ren 6421	PE	PE00865787	19580802
Xue-Gang Sun et al. 2457	PE	PE01801030	20010721
Yong-Tian Zhang & Kai-Yong Lang 975	PE	PE00865823	19650727
You-Wen Cui 5280	PE	PE00865801	19510812
Ze-Sun Yu et al. 06451	PE	PE00865776	19740705
Ze-Yun Zhang & Hong-Fu Zhou 22893	PE	PE00865782	19570717
Ze-Yun Zhang & Hong-Fu Zhou 22893	PE	PE00865807	19570717
Zhen-Shu Liu 1095	PE	PE00865803	19340000
Zhi-Ping Huang et al. 1671	PE	PE00865804	19300807
Zhong-Tian Guan 450168	PE	PE00865793	19600804
Zhong-Tian Guan 460462	PE	PE00865775	19600802
Zhu He & Zi-Lin Zhou 13449	PE	PE00865809	19520000
Zhu He & Zi-Lin Zhou 13671	PE	PE01982468	19520000
Zuo-Bin Wang 14542	PE	PE00865906	19510722
BeiChuan Team 38	SM	SM706501164	19780722
General Investigation Team 664	SM	SM	19800813
General Investigation Team 664	SM	SM706501157	19800813
KangDing Team 664	SM	SM	19800808
KangDing Team 664	SM	SM706501167	19800808
Li Team 196	SM	SM706501165	19790712
Li Team 217	SM	SM706501168	19790720
MaoWen Group 1104	SM	SM	19790719
MaoWen Group 1104	SM	SM706501155	19790719
PingWu Team 648	SM	SM706501166	19780721
Shan-Yong Chen et al. 5327	SM	SM706501158	19580626
Shan-Yong Chen et al. 5702	SM	SM	19580801
Shan-Yong Chen et al. 5702	SM	SM706501159	19580801
TianJin General Investigation Team 78-562	SM	SM706501163	19780801
Unknown Collector 132	SM	SM	19790710
Unknown Collector 198	SM	SM	19790729
Unknown Collector 22352	SM	SM706501153	19600801
Unknown Collector 22457	SM	SM706501154	19600814
Unknown Collector 22774	SM	SM706501152	19600816

Unknown Collector 405	SM	SM	19790903
Unknown Collector 423	SM	SM	19780824
Unknown Collector 500	SM	SM	19780824
Unknown Collector 500	SM	SM706501161	19780804
Unknown Collector 5327	SM	SM	
Unknown Collector 5327	SM	SM	
Unknown Collector 805	SM	SM	19790721
Unknown Collector 805	SM	SM706501156	19790721
Unknown Collector 817	SM	SM	19790719
Unknown Collector 832	SM	SM	19790716
Unknown Collector 832	SM	SM706501189	19790716
Unknown Collector 848	SM	SM	19790716
Unknown Collector 848	SM	SM706501190	19790716
YaJiang Team 298	SM	SM	19790809
YaJiang Team 298	SM	SM706501172	19790809
Yuan Han 599	SM	SM706501162	19780919
Hai-Xia Ye	SZ	SZ00377568	20070704
Hong-Fu Zhou 22893	SZ	SZ00179789	19570717
Hong-Fu Zhou 22893	SZ	SZ00179802	19570717
Jun-Chao Gu 1072	SZ	SZ00377557	20070705
NanShuiBeiDiao Team 01535	SZ	SZ00377534	19590703
Ninth Group 1242	SZ	SZ00377605	20070705
Qing-Sheng Zhao 125963	SZ	SZ00431951	19890801
Qing-Sheng Zhao 125963	SZ	SZ00431952	19890801
Qing-Sheng Zhao 125963	SZ	SZ00431953	19890801
Qing-Sheng Zhao 125963	SZ	SZ00431954	19890801
Qing-Sheng Zhao 125963	SZ	SZ00431955	19890801
Qing-Sheng Zhao 125963	SZ	SZ00431956	19890801
Qing-Sheng Zhao 125963	SZ	SZ00431957	19890801
Qing-Sheng Zhao 125963	SZ	SZ00431958	19890801
Qing-Sheng Zhao 6576	SZ	SZ00179798	19780813
Qing-Sheng Zhao 6576	SZ	SZ00179799	19780813
Qing-Sheng Zhao 6576	SZ	SZ00179800	19780813
Qing-Sheng Zhao 6576	SZ	SZ00179850	19780813
Shan-Yong Chen et al. 5327	SZ	SZ00179788	19580626
Shan-Yong Chen et al. 5702	SZ	SZ00179851	19580801
Third Group 20070339	SZ	SZ00377601	20070704
Trainee 03-3-1090	SZ	SZ00377556	20060701
Unknown Collector	SZ	SZ00179801	
Unknown Collector	SZ	SZ00179944	
Unknown Collector 02272	SZ	SZ00179785	19590722
Unknown Collector 02272	SZ	SZ00179787	19590722
Unknown Collector 03-3-1090	SZ	SZ00377600	
Unknown Collector 03-3-1097	SZ	SZ00377529	

Unknown Collector 0832	SZ	SZ00180218	19790716
Unknown Collector 0832	SZ	SZ00180219	19790716
Unknown Collector 0896	SZ	SZ00180209	19790719
Unknown Collector 122556	SZ	SZ00180231	19870802
Unknown Collector 122556	SZ	SZ00180232	19870802
Unknown Collector 45-0168	SZ	SZ00179784	19600804
Unknown Collector 7	SZ	SZ00377526	20010710
Unknown Collector 789	SZ	SZ00180079	
Unknown Collector 805	SZ	SZ00180221	19790721
Unknown Collector 805	SZ	SZ00180224	19790721
Unknown Collector WL0138	SZ	SZ00377603	20050709
Wen-Guang Hu & Zhu He 10922	SZ	SZ00179797	19510818
Wen-Guang Hu & Zhu He 10972	SZ	SZ00179804	19510820
Wen-Pei Fang & Ze-Rong Zhang 22504	SZ	SZ00179786	19570702
Wen-Pei Fang et al. 22435	SZ	SZ00179803	19570628
Wen-Pei Fang et al. 22435	SZ	SZ00179858	19570628
Wen-Pei Fang et al. 22504	SZ	SZ00179808	19570702
Wen-Pei Fang et al. 22593	SZ	SZ00179790	19570627
Wen-Pei Fang et al. 22593	SZ	SZ00179791	19570627
Xin Li & Ji-Xi Zhou 73917	SZ	SZ00179792	19570732
Xin Li & Ji-Xi Zhou 73917	SZ	SZ00179807	19570731
Xin Li 71548	SZ	SZ00179806	19570618
Xin Li 73027	SZ	SZ00179849	19570708
Xin Li 73027	SZ	SZ00179855	19570708
Xing-Jin He & Qing-Sheng Zhao	SZ	SZ00431936	20080716
Xing-Jin He & Qing-Sheng Zhao	SZ	SZ00431945	20080716
Xing-Jin He & Qing-Sheng Zhao	SZ	SZ00431946	20080714
Xing-Jin He & Qing-Sheng Zhao	SZ	SZ00431947	20080716
Xing-Jin He & Qing-Sheng Zhao	SZ	SZ00431948	20080715
Xing-Jin He & Qing-Sheng Zhao	SZ	SZ00431949	20080715
Xing-Jin He & Qing-Sheng Zhao 187568	SZ	SZ00431937	20080714
Xing-Jin He & Qing-Sheng Zhao 187568	SZ	SZ00431938	20080714
Xing-Jin He & Qing-Sheng Zhao 187568	SZ	SZ00431944	20080714
Xing-Jin He & Qing-Sheng Zhao 188730	SZ	SZ00431935	20080716
Xing-Jin He & Qing-Sheng Zhao 189729	SZ	SZ00431934	20080714
Xing-Jin He & Qing-Sheng Zhao 189729	SZ	SZ00431941	20080714

Xing-Jin He & Qing-Sheng Zhao 189729	SZ	SZ00431942	20080714
Xing-Jin He & Qing-Sheng Zhao 189729	SZ	SZ00431943	20080714
Xing-Jin He & Qing-Sheng Zhao 189888	SZ	SZ00431932	20080716
Xing-Jin He & Qing-Sheng Zhao 189888	SZ	SZ00431933	20080716
Xing-Jin He & Qing-Sheng Zhao 189888	SZ	SZ00431939	20080716
Xing-Jin He & Qing-Sheng Zhao 189888	SZ	SZ00431940	20080716
Xing-Jin He et al. 143731	SZ	SZ00377592	20070729
Xing-Jin He et al. 143731	SZ	SZ00377593	20070729
Xing-Jin He et al. 143731	SZ	SZ00377594	20070729
Xing-Jin He et al. 143731	SZ	SZ00377606	20070729
Xing-Jin He et al. 143919	SZ	SZ00377581	20070810
Xing-Jin He et al. 143919	SZ	SZ00377582	20070810
Xing-Jin He et al. 143919	SZ	SZ00377583	20070810
Xing-Jin He et al. 143919	SZ	SZ00377584	20070810
Xing-Jin He et al. 146385	SZ	SZ00377559	20070810
Xing-Jin He et al. 146385	SZ	SZ00377560	20070810
Xing-Jin He et al. 146445	SZ	SZ00377574	20070731
Xing-Jin He et al. 146445	SZ	SZ00377575	20070731
Xing-Jin He et al. 146445	SZ	SZ00377576	20070731
Xing-Jin He et al. 146445	SZ	SZ00377577	20070731
Xing-Jin He et al. 146695	SZ	SZ00377598	20070731
Xing-Jin He et al. 146695	SZ	SZ00377599	20070731
Xing-Jin He et al. 147996	SZ	SZ00377570	20070731
Xing-Jin He et al. 147996	SZ	SZ00377571	20070731
Xing-Jin He et al. 147996	SZ	SZ00377572	20070731
Xing-Jin He et al. 147996	SZ	SZ00377573	20070731
Xing-Jin He et al. 149951	SZ	SZ00377561	20070810
Xing-Jin He et al. 149951	SZ	SZ00377562	20070810
Xing-Jin He et al. 149951	SZ	SZ00377563	20070810
Xing-Jin He et al. 149951	SZ	SZ00377564	20070810
Xing-Jin He et al. 150125	SZ	SZ00377565	20070810
Xing-Jin He et al. 150125	SZ	SZ00377566	20070810
Xing-Jin He et al. 150125	SZ	SZ00377567	20070810
Xing-Jin He et al. 150125	SZ	SZ00377569	20070810
Xing-Jin He et al. 150903	SZ	SZ00377530	20070809
Xing-Jin He et al. 150903	SZ	SZ00377531	20070809
Xing-Jin He et al. 150903	SZ	SZ00377532	20070809
Xing-Jin He et al. 150903	SZ	SZ00377553	20070809

Xing-Jin He et al. 150903	SZ	SZ00377595	20070809
Xing-Jin He et al. 150903	SZ	SZ00377596	20070809
Xing-Jin He et al. 150903	SZ	SZ00377597	20070809
Xing-Jin He et al. 150960	SZ	SZ00377585	20070809
Xing-Jin He et al. 150960	SZ	SZ00377586	20070809
Xing-Jin He et al. 150960	SZ	SZ00377587	20070809
Xing-Jin He et al. 150960	SZ	SZ00377588	20070812
Xing-Jin He et al. 150960	SZ	SZ00377589	20070812
Xing-Jin He et al. 150960	SZ	SZ00377590	20070812
Xing-Jin He et al. 150960	SZ	SZ00377591	20070812
Xing-Jin He et al. 153245	SZ	SZ00377554	20070809
Xing-Jin He et al. 153245	SZ	SZ00377555	20070809
Xing-Jin He et al. 153355	SZ	SZ00377578	20070811
Xing-Jin He et al. 153355	SZ	SZ00377579	20070811
Xing-Jin He et al. 153355	SZ	SZ00377580	20070811
Xiu-Shi Zhang & You-Xian Ren 5941	SZ	SZ00179795	19580717
Xiu-Shi Zhang & You-Xian Ren 6004	SZ	SZ00179794	19580720
Xiu-Shi Zhang & You-Xian Ren 6004	SZ	SZ00179854	19580720
Xiu-Shi Zhang & You-Xian Ren 6348	SZ	SZ00179793	19580728
Xiu-Shi Zhang & You-Xian Ren 6421	SZ	SZ00179853	19580802
Yong Sun 2007-057	SZ	SZ00377602	20070704
Yu Fan 1082	SZ	SZ00377558	20070705
Ze-Rong Zhang & Hong-Fu Zhou 23602	SZ	SZ00179805	19570810
Zhen-Shu Liu 1095	SZ	SZ00179796	
Zhong-Ming Tan & Shu-Hua Yu 122550	SZ	SZ00180258	19870802
Zhong-Ming Tan & Shu-Hua Yu 122550	SZ	SZ00180259	19870802
Zhong-Ming Tan & Shu-Hua Yu 122550	SZ	SZ00180260	19870802
Zhong-Ming Tan & Shu-Hua Yu 122556	SZ	SZ00180257	19870802
Zhong-Ming Tan & Shu-Hua Yu 122557	SZ	SZ00180159	19870802
Zhong-Ming Tan & Shu-Hua Yu 122557	SZ	SZ00180160	19870802
Zhong-Ming Tan & Shu-Hua Yu 122557	SZ	SZ00180162	19870802

Zhong-Ming Tan & Shu-Hua Yu 122579	SZ	SZ00180133	19870803
Zhong-Ming Tan & Shu-Hua Yu 122579	SZ	SZ00180136	19870803
Zhong-Ming Tan & Shu-Hua Yu 122579	SZ	SZ00180207	19870803
Zhong-Ming Tan & Shu-Hua Yu 122588	SZ	SZ00180134	19870803
Zhong-Ming Tan & Shu-Hua Yu 122588	SZ	SZ00180135	19870803
Zhong-Ming Tan & Shu-Hua Yu 122589	SZ	SZ00180247	19870803
Zhong-Ming Tan & Shu-Hua Yu 123001	SZ	SZ00180244	19870804
Zhong-Ming Tan & Shu-Hua Yu 123001	SZ	SZ00180245	19870804
Zhong-Ming Tan & Shu-Hua Yu 123001	SZ	SZ00180246	19870804
Zhong-Ming Tan & Shu-Hua Yu 123002	SZ	SZ00180239	19870804
Zhong-Ming Tan & Shu-Hua Yu 123003	SZ	SZ00180132	19870804
Zhong-Ming Tan & Shu-Hua Yu 123003	SZ	SZ00180235	19870804
Zhong-Ming Tan & Shu-Hua Yu 123003	SZ	SZ00180236	19870804
Zhong-Ming Tan & Shu-Hua Yu 123003	SZ	SZ00180238	19870804
Zhong-Ming Tan & Shu-Hua Yu 123004	SZ	SZ00180141	19870804
Zhong-Ming Tan & Shu-Hua Yu 123004	SZ	SZ00180230	19870804
Zhong-Ming Tan & Shu-Hua Yu 123004	SZ	SZ00180254	19870804
Zhong-Ming Tan & Shu-Hua Yu 123005	SZ	SZ00180126	19870804
Zhong-Ming Tan & Shu-Hua Yu 123005	SZ	SZ00180127	19870804
Zhong-Ming Tan & Shu-Hua Yu 123005	SZ	SZ00180204	19870804
Zhong-Ming Tan & Shu-Hua Yu 123052	SZ	SZ00180122	19870806
Zhong-Ming Tan & Shu-Hua Yu 123052	SZ	SZ00180123	19870806

Zhong-Ming Tan & Shu-Hua Yu 123052	SZ	SZ00180124	19870806
Zhong-Ming Tan & Shu-Hua Yu 123063	SZ	SZ00180163	19870806
Zhong-Ming Tan & Shu-Hua Yu 123063	SZ	SZ00180164	19870806
Zhong-Ming Tan & Shu-Hua Yu 123063	SZ	SZ00180165	19870806
Zhong-Ming Tan & Shu-Hua Yu 123075	SZ	SZ00180155	19870807
Zhong-Ming Tan & Shu-Hua Yu 123075	SZ	SZ00180156	19870807
Zhong-Ming Tan & Shu-Hua Yu 123075	SZ	SZ00180157	19870807
Zhong-Ming Tan & Shu-Hua Yu 123364	SZ	SZ00431929	19870828
Zhong-Ming Tan & Shu-Hua Yu 123364	SZ	SZ00431930	19870828
Zhong-Ming Tan 123003	SZ	SZ00180121	19870804
Zhong-Wei Feng 20070916	SZ	SZ00377604	20070705
Zhu He 13449	SZ	SZ00179852	19520723
Zhu He 13671	SZ	SZ00179965	19520816
Parnassia delavayi			
Ce Shang I"-222	BJFC	BJFC	20130726
Jin-Yu Li & Jin Zhang SN201107042	BJFC	BJFC	20110812
Lei Xie EM07	BJFC	BJFC	20150901
Lei Xie EM29	BJFC	BJFC	20150901
Li He & Jun Zhao HL36	BJFC	BJFC	20160714
Li He & Xing-Xing Mao PH20120614-05	BJFC	BJFC	20120614
Li He et al. PH20120625-11	BJFC	BJFC	20120625
Li He et al. PH20120716-02	BJFC	BJFC	20120716
Li He et al. PH20120802-16	BJFC	BJFC	20120802
Xing-Xing Mao 2013072923	BJFC	BJFC	20130729
Yu-Min Shu & Feng-Bin Zhao sz003	BJFC	BJFC	20140729
Yu-Min Shu & Feng-Bin Zhao sz006	BJFC	BJFC	20140729
Yu-Min Shu & Feng-Bin Zhao sz028	BJFC	BJFC	20140729
Yu-Min Shu & Feng-Bin Zhao sz030	BJFC	BJFC	20140729

Yu-Min Shu & Rong-Yan Deng sd703	BJFC	BJFC	20151013
Yu-Ming Shu & Lei Wang sw361	BJFC	BJFC	20150801
Yu-Ming Shu & Lei Wang sw364	BJFC	BJFC	20150801
Yu-Ming Shu & Lei Wang sw367	BJFC	BJFC	20150801
Yu-Ming Shu & Lei Wang sw372	BJFC	BJFC	20150801
Yu-Ming Shu & Lei Wang sw414	BJFC	BJFC	20150803
Yu-Ming Shu & Lei Wang sw417	BJFC	BJFC	20150803
Yu-Ming Shu & Lei Wang sw418	BJFC	BJFC	20150803
Yu-Ming Shu & Lei Wang sw423	BJFC	BJFC	20150803
Yu-Ming Shu & Lei Wang sw437	BJFC	BJFC	20150805
Yu-Ming Shu & Lei Wang sw438	BJFC	BJFC	20150805
Yu-Ming Shu & Lei Wang sw439	BJFC	BJFC	20150805
Yu-Ming Shu & Lei Wang sw497	BJFC	BJFC	20150812
Yu-Ming Shu & Lei Wang sw498	BJFC	BJFC	20150812
Yu-Ming Shu & Lei Wang sw527	BJFC	BJFC	20150816
Guang-Hui Yang 59726	CDBI	CDBI0039573	19581009
Qing-Sheng Zhao et al. 6736	CDBI	CDBI0039161	19780723
Qing-Sheng Zhao et al. 6736	CDBI	CDBI0039162	19780723
Qing-Sheng Zhao et al. 6736	CDBI	CDBI0039163	19780723
Qing-Sheng Zhao et al. 6840	CDBI	CDBI0039727	19780728
Qing-Sheng Zhao et al. 6840	CDBI	CDBI0038364	19780728
Qing-Sheng Zhao et al. 6840	CDBI	CDBI0038365	19780728
Qing-Sheng Zhao et al. 7885	CDBI	CDBI0038500	19780731
Qing-Sheng Zhao et al. 7885	CDBI	CDBI0038501	19780731
Qing-Sheng Zhao et al. 7885	CDBI	CDBI0039155	19780731
Qing-Sheng Zhao et al. 7885	CDBI	CDBI0039156	19780731
Tai-Chang 20612	CDBI	CDBI0038594	19790717
Tibet Team 2189	CDBI	CDBI0038741	19810706
Tibet Team 2189	CDBI	CDBI0039420	19810706
Tibet Team 2254	CDBI	CDBI0039307	19810709
Tibet Team 2254	CDBI	CDBI0038431	19810709
Tibet Team 2308	CDBI	CDBI0039185	19810710
Tibet Team 2308	CDBI	CDBI0039431	19810710
Tibet Team 2518	CDBI	CDBI0039088	19810709
Tibet Team 2518	CDBI	CDBI0039396	19810709
Unknown Collector 1568	CDBI	CDBI0039056	19590802
Unknown Collector 1568	CDBI	CDBI0039561	19590802
Unknown Collector 4778	CDBI	CDBI0039723	19740704
Unknown Collector 4778	CDBI	CDBI0039724	19740704
Unknown Collector 4778	CDBI	CDBI0039569	19740704
Vegetation Group 31397	CDBI	CDBI0037155	19830820
Vegetation Group 31397	CDBI	CDBI0037156	19830820
Vegetation Group 31397	CDBI	CDBI0037157	19830820

Ya-Bin Yang 7323	CDBI	CDBI0039582	19780711
Ya-Bin Yang 7323	CDBI	CDBI0039583	19780711
Ya-Bin Yang 7323	CDBI	CDBI0039154	19780711
Yu-Rong Xu 23439	CDBI	CDBI0037158	19800911
Yu-Rong Xu 23439	CDBI	CDBI0037151	19800911
Ze-Rong Zhang et al. 124	CDBI	CDBI0037159	19630814
Ze-Rong Zhang et al. 124	CDBI	CDBI0037160	19630814
Zhen-Ju Zhao et al. 114897	CDBI	CDBI0039595	19810711
Zhen-Ju Zhao et al. 115239	CDBI	CDBI0039594	19810817
Ai-Hua Li 000157	KUN	KUN0768620	20060915
Cai-Qi Li 3242	KUN	KUN0437203	19400810
Cai-Qi Li 3954	KUN	KUN0437207	19400810
China & Germany Team 88-064	KUN	KUN0437115	19880724
Chong-Yun Zhao 20804	KUN	KUN0437108	19390723
Chong-Yun Zhao 20804	KUN	KUN0437109	19390723
Chong-Yun Zhao 20804	KUN	KUN0437110	19390723
Cong-Li Yang 75-15	KUN	KUN0437127	19750909
De-Ding Tao et al. 1002	KUN	KUN0437111	19820908
De-Jun Yu 12079	KUN	KUN0437078	19370709
De-Jun Yu 12194	KUN	KUN0437073	19370716
De-Jun Yu 12194	KUN	KUN0437072	19370716
De-Jun Yu 15397	KUN	KUN0437074	19370803
De-Jun Yu 15397	KUN	KUN0437075	19370803
De-Jun Yu 16946	KUN	KUN0437088	19380724
De-Jun Yu 16946	KUN	KUN0437089	19380724
De-Jun Yu 22207	KUN	KUN0437090	19380806
De-Jun Yu 22207	KUN	KUN0437091	19380806
De-Jun Yu 22402	KUN	KUN0437079	19380818
De-Jun Yu 22493	KUN	KUN0437082	19380826
De-Jun Yu 22493	KUN	KUN0437083	19380826
De-Jun Yu 22600	KUN	KUN0437080	19380831
De-Jun Yu 22600	KUN	KUN0437081	19380831
De-Jun Yu 22782	KUN	KUN0437084	19381008
De-Jun Yu 22782	KUN	KUN0437085	19381008
De-Jun Yu 6992	KUN	KUN0437189	19370711
De-Jun Yu 7603	KUN	KUN0437190	19370805
De-Jun Yu 8894	KUN	KUN0437093	19370711
De-Jun Yu 8971	KUN	KUN0437092	19370714
De-Jun Yu 8971	KUN	KUN0437094	19370714
De-Jun Yu 9787	KUN	KUN0437076	19370823
De-Jun Yu 9787	KUN	KUN0437077	19370823
Department of biology, Yunnan University 229	KUN	KUN0437157	19560705
Dian North-East Group 407	KUN	KUN0437130	19640726

Dian North-East Group 407	KUN	KUN0437133	19640726
Dian North-East Group 511	KUN	KUN0437131	19640813
Dian North-East Group 511	KUN	KUN0437132	19640813
Dian North-West JinSha River Team 63-6124	KUN	KUN0437134	19630725
Dian North-west JinSha River Team 63-6124	KUN	KUN0437135	19630725
Ding Wu & Jin-Mei Lu 3074	KUN-1	KUN-1	20030724
Ding WU & Shu-Dong Zhang 5011	KUN-1	KUN-1	20050814
Ding Wu et al. 2004	KUN-1	KUN-1	20020826
DuLongJiang Research Team 7041	KUN	KUN0437095	19910522
DuLongJiang Research Team 7041	KUN	KUN0437096	19910522
En-De Liu 5057	KUN	KUN0491260	20020712
En-De Liu 5057	KUN	KUN0491268	20020712
En-De Liu 6278	KUN	KUN0491207	20020813
En-De Liu 6278	KUN	KUN0491258	20020813
En-De Liu 6301	KUN	KUN0491257	20020813
En-De Liu et al. 2193	KUN	KUN0774512	20090806
Fan-Jin Dai 5247	KUN	KUN0437191	19390927
Fa-Zuan Wang 23402	KUN	KUN0437188	19310719
Guang-Hui Yang 59726	KUN	KUN0437208	19581009
Guo-Feng Li 63377	KUN	KUN0437209	19570000
Guo-Feng Li 83836	KUN	KUN0437210	19570000
Guo-Mei Feng 1621	KUN	KUN0437170	19390712
Guo-Mei Feng 1621	KUN	KUN0437172	19390712
Guo-Mei Feng 2081	KUN	KUN0437171	19390820
Guo-Mei Feng 2081	KUN	KUN0437173	19390820
Guo-Mei Feng 21289	KUN	KUN0437174	19550901
Guo-Mei Feng 21289	KUN	KUN0437175	19550901
Guo-Mei Feng 22526	KUN	KUN0437176	19590806
Guo-Mei Feng 22526	KUN	KUN0437177	19590806
Guo-Mei Feng 22526	KUN	KUN0437178	19590806
Guo-Mei Feng 2890	KUN	KUN0437204	19390831
Guo-Mei Feng 2890	KUN	KUN0437206	19390831
Guo-Mei Feng 5573	KUN	KUN0437166	19400718
Guo-Mei Feng 5573	KUN	KUN0437168	19400718
Guo-Mei Feng 5892	KUN	KUN0437167	19400803
Guo-Mei Feng 5892	KUN	KUN0437169	19400803
H Li 0433	KUN	KUN0137238	20020816
H Li 0433	KUN	KUN1307591	20020816
Hong Wang et al. 32088	KUN-1	KUN-1	20020826

Hong Wang et al. 894	KUN	KUN0679518	20030817
Hua Peng et al. 5024	KUN	KUN0137479	20030826
Hua Peng et al. 5024	KUN	KUN0137480	20030826
Hua Peng et al. 8516	KUN	KUN0773443	20080728
Hua Peng et al. 8516	KUN	KUN0773444	20080728
Hua Peng et al. 8870	KUN	KUN0773441	20080730
Hua Peng et al. 8870	KUN	KUN0773442	20080730
Jin-Mei Lu 3054	KUN-1	KUN-1	20030727
Ji-Pei Yue 3084	KUN-1	KUN-1	20030826
JM Delavay	KUN	KUN1217507	18830724
Kun-Jun Fu 8337	KUN	KUN0437186	19560731
LianDa 11455	KUN	KUN0437086	19380827
LianDa 21124	KUN	KUN0437087	
Lian-MIng Gao & Jin-Mei Lu 2317	KUN-1	KUN-1	20030819
Lian-Ming Gao 103061	KUN	KUN1242202	20100824
Lin-Han Liu 010601	KUN	KUN0437181	19640802
M Iabbe Delavay	KUN	KUN1221634	18900719
Na-Na Lin & Wen-Bin Yu 1025	KUN-1	KUN-1	20050708
NanShuiBeiDiao Team 9796	KUN	KUN0437153	19600707
NanShuiBeiDiao Team 9796	KUN	KUN0437154	19600707
PanGong Team 2084	KUN-1	KUN-1	20020912
Pei-Yuan Li 8589	KUN	KUN0437187	19560730
Ping-Hua Yu 786	KUN	KUN0437179	19570913
Ping-Hua Yu 786	KUN	KUN0437180	19570913
Ping-Hua Yu 963	KUN	KUN0437155	19570927
Ping-Hua Yu 963	KUN	KUN0437156	19570927
Qin Lin 791988	KUN	KUN0437123	19790801
Qin Lin 791988	KUN	KUN0437124	19790801
Qin Lin et al. 771689	KUN	KUN0437241	1985004
Qin Lin et al. 771689	KUN	KUN0437242	1985004
Qi-Wu Wang 64600	KUN	KUN0437145	19350700
Qi-Wu Wang 68534	KUN	KUN0437146	19350800
Qi-Wu Wang 68630	KUN	KUN0437147	19350800
Qi-Wu Wang 68768	KUN	KUN0437148	19350800
Ren-Chang Qin 20994	KUN	KUN0437104	19390715
Ren-Chang Qin 20994	KUN	KUN0437105	19390715
Ren-Chang Qin 21187	KUN	KUN0437106	19380731
Ren-Chang Qin 21187	KUN	KUN0437107	19380731
Ren-Chang Qin 23419	KUN	KUN0437101	19290729
Ren-Chang Qin 23419	KUN	KUN0437102	19290729
Ren-Chang Qin 24016	KUN	KUN0437103	19290823
Ren-Chang Qin 24370	KUN	KUN0437099	19290906
Ren-Chang Qin 24370	KUN	KUN0437100	19290906

Ren-Chang Qin 30441	KUN	KUN0437097	19390725
Ren-Chang Qin 30441	KUN	KUN0437098	19390725
RP Farges 29	KUN	KUN1230258	
RP Maire	KUN	KUN1225519	19210810
RP Maire	KUN	KUN1230234	19140815
Sheng-Tang Li 80-283	KUN	KUN0437125	19800726
Sheng-Tang Li 80-283	KUN	KUN0437126	19800726
Sino-US Joint Western Hubei Plant research Team 37	KUN	KUN0437182	19800824
Sino-US Joint Western Hubei Plant research Team 37	KUN	KUN0437183	19800824
Tibet Team 13052	KUN	KUN0437192	19830819
Tibet Team 13052	KUN	KUN0437194	19830819
Tibet Team 13174	KUN	KUN0437193	19830821
Tibet Team 13174	KUN	KUN0437195	19830821
Tibet Team 2189	KUN	KUN0437112	19810706
Tibet Team 2189	KUN	KUN0437120	19810707
Tibet Team 2254	KUN	KUN0437121	19810709
Tibet Team 2254	KUN	KUN0437122	19810709
Tibet Team 2308	KUN	KUN0437116	19810710
Tibet Team 2308	KUN	KUN0437117	19810710
Tibet Team 2518	KUN	KUN0437118	19810709
Tibet Team 2518	KUN	KUN0437119	19810709
Tibet Team 8556	KUN	KUN0437136	19820725
Tibet Team 8556	KUN	KUN0437137	19820725
Unknown Collector	KUN	KUN0437198	19820903
Unknown Collector	KUN	KUN0437212	
Unknown Collector 1212	KUN	KUN0437211	
Unknown Collector 1568	KUN	KUN0437200	19590802
Unknown Collector 1853	KUN	KUN0437199	19590821
Unknown Collector 2138	KUN	KUN0437202	
Unknown Collector 3823	KUN	KUN0437201	19590712
Unknown Collector 489	KUN	KUN0437138	
Wen-Cai Wang 100	KUN	KUN0437151	19580902
Wen-Cai Wang 100	KUN	KUN0437152	19580902
Xi-Tao Cai 52019	KUN	KUN0437141	19320913
Xi-Tao Cai 52019	KUN	KUN0437143	19320913
Xi-Tao Cai 53724	KUN	KUN0437142	19330817
Xi-Tao Cai 53724	KUN	KUN0437144	19330817
Xi-Tao Cai 53860	KUN	KUN0437139	19330727
Xi-Tao Cai 53860	KUN	KUN0437140	19330727
Ying Jiang 11510	KUN	KUN0437149	19330400
Ying Jiang 11510	KUN	KUN0437150	19330400
Ying Jiang 11510	KUN	KUN0437449	19330000

ZD Fang 990	KUN	KUN0437114	19880717
Zheng-Yi Wu 4279	KUN	KUN0437128	19760808
Zheng-Yi Wu 4279	KUN	KUN0437129	19760808
Zheng-Yi Wu et al. 86-289	KUN	KUN0437213	19860923
Zheng-Yi Wu et al. 88	KUN	KUN0437196	19800825
Zheng-Yi Wu et al. 88	KUN	KUN0437197	19800825
Zheng-Yu Liu 782625	IMC	IMC000014623	19780812
Zheng-Yu Liu 782625	IMC	IMC000014624	19780812
Zheng-Yu Liu 782625	IMC	IMC000014625	19780812
Zheng-Yu Liu 782625	IMC	IMC000014658	19780812
Zhi-Dan Wei et al. 5735	KUN	KUN1342383	20170801
Zhi-Jian Yin et al. 1265	KUN	KUN1215329	20090715
Zhi-Jian Yin et al. 1265	KUN	KUN1215479	20090715
ZhongDian Team 1385	KUN	KUN0437159	19620826
ZhongDian Team 1385	KUN	KUN0437161	19620826
ZhongDian Team 1965	KUN	KUN0437162	19620909
ZhongDian Team 1965	KUN	KUN0437163	19620909
ZhongDian Team 63-3415	KUN	KUN0437164	19630714
ZhongDian Team 63-3415	KUN	KUN0437165	19630714
ZhongDian Team 850	KUN	KUN0437158	19620816
ZhongDian Team 850	KUN	KUN0437160	19620816
Zuo-Bin Wang 15667	KUN	KUN0437185	19520708
Zuo-Bin Wang 16437	KUN	KUN0437184	19520903
A Monbeig	P	P03331424	1908
Delavay	P	P05556320	18890914
Delavay	P	P05556321	18890914
Delavay	P	P05583619	18890719
Delavay	P	P05583623	
Delavay	P	P06392627	18880729
Delavay	P	P06392628	18880729
Delavay	P	P06392629	18880729
Delavay	P	P06392633	18890904
Delavay	P	P06392635	18850911
Delavay	P	P06392668	18840901
Delavay 1	P	P05556380	18830724
Delavay 1	P	P05556385	18830724
Delavay 1	P	P06392626	18830724
Delavay 1	P	P06392632	
Delavay 130	P	P06392623	
Delavay 2	P	P06392622	18920700
Delavay 217	P	P05556394	18830816
Delavay 217	P	P05556395	18830816
Delavay 217	P	P06392624	
Delavay 72	P	P05494801	18840823

Delavay 72	P	P06392630	18840823
Delavay 72	P	P06392634	18840823
EH Wilson 4089	P	P05583613	
EH Wilson 4895	P	P03331598	19030000
Farges	P	P05556384	
Farges	P	P05556389	
Farges	P	P05583580	
Farges	P	P05583581	
Farges 27	P	P05494800	
Farges 27	P	P05556393	
Farges 27	P	P06392655	
Farges 27	P	P06392661	
Farges 27	P	P06392662	
Farges 27	P	P06392646	
Forrest 6196	P	P06392657	19100700
Fr Ducloux 4315	P	P03371245	19060800
Fr Ducloux 4315	P	P03371246	19060800
FT Wang	P	P03370640	1931
Henry 6542	P	P06392636	18890000
Henry 6542	P	P06392641	18890000
Henry 7260	P	P06392642	18890000
JM Delavay 130	P	P00709357	1883-8-7
JM Delavay 130	P	P00709358	1883-8-7
JM Delavay 217	P	P00709355	1883-8-16
JM Delavay 217	P	P00709356	1883-8-16
M Fr Ducloux 6407	P	P03331419	190907
Maire	P	P04446728	
Maire	P	P05608135	
McLaren 79	P	P06392639	19380000
Monbeig	P	P06865506	
Rock 16949	P	P06392638	19280800
Rock 18500	P	P06392650	19290700
Rock 5796	P	P06392648	19220000
Soulie	P	P06392640	
Soulie 13	P	P06392645	
Soulie 1304	P	P06865510	
Soulie 304	P	P06392647	
Soulie 304	P	P06392644	
Soulie 793	P	P05494769	
Soulie 818	P	P05608125	18930000
Soulie 818	P	P06392659	1893
Soulie 818	P	P06392660	1893
Unknown Collector	P	P06392658	
Unknown Collector 27	P	P06392654	

Wen-Pei Fang 2785	P	P06392651	19280811
Aug Henry 7260	PE	PE01982470	
BaShan Collection Team 2286	PE	PE01857424	20080725
BaShan Collection Team 2499	PE	PE01857425	20080726
Ben-Zhao Guo 899	PE	PE00865896	19510901
Cai-Fei Zhang 1061	PE	PE	20080727
Cai-Fei Zhang 1061	PE	PE	20080727
Cong-Rong Wang 1005	PE	PE01869782	19930713
CS Liu 822	PE	PE00865941	19340723
Da-Hua Du 589	PE	PE00865956	19350813
De-Jun Yu 3931	PE	PE00865951	19340831
Delavay	PE	PE	18830724
Dian North-west JinSha River Group 63-6124	PE	PE00865967	19630725
Dian North-west JinSha River Group 63-6124	PE	PE00865968	19630725
Dr Aug Henry 6542	PE	PE00865914	
Fa-Zuan Wang et al. 086	PE	PE00865895	19551003
Fa-Zuan Wang et al. 144	PE	PE00861986	19550929
feng 2081	PE	PE01982466	19390820
FT Wang 23402	PE	PE00865988	19310722
FT Wang 23402	PE	PE00866144	19310722
Fu-Ming Zhao 21187	PE	PE00866004	19390731
G Forrest 12487	PE	PE00866060	19140000
G Forrest 28537	PE	PE00866059	19300000
G Forrest 29147	PE	PE00866062	19300000
G Forrest 6196	PE	PE00866058	19100000
G.Forrest 28086	PE	PE00866061	19290400
General Investigation 14523	PE	PE00866730	19590000
General Investigation Specimen 20346	PE	PE00865881	19590000
General Investigation Specimen 20346	PE	PE00865882	19590000
Guang-Hui Yang 56455	PE	PE00865931	19590806
Guang-Hui Yang 56455	PE	PE00865954	19590806
Guang-Hui Yang 56633	PE	PE00866141	19570813
Guang-Hui Yang 57412	PE	PE00865930	19570926
Guang-Hui Yang 58765	PE	PE00861988	19580714
Guang-Hui Yang 58765	PE	PE00865916	19580714
Guang-Hui Yang 59728	PE	PE00865908	19581009
Guang-Yu Hong 4063	PE	PE02059342	10200713
Guang-Zhao Li 15542	PE	PE00862032	19970725
Gui-Ling Qu 3524	PE	PE00865959	19360000
Gui-Ling Qu 3524	PE	PE00865961	19360000

Gui-Ling Qu 3524	PE	PE00865963	19360000
Guo-Feng Li 63877	PE	PE00866142	19570829
Guo-Mei Feng 21289	PE	PE00865974	19550901
Guo-Xun Fu & Zhi-Song Zhang 1512	PE	PE00865909	19570903
Guo-Xun Fu & Zhi-Song Zhang 1512	PE	PE00865910	19570903
Guo-Xun Fu & Zhi-Song Zhang 1512	PE	PE00865911	19570903
Han-Chen Wang 1206	PE	PE00865981	19410800
Han-Chen Wang 4497	PE	PE00866007	19440813
HeNan Team 1233	PE	PE00865883	19600718
HeNan Team 2529	PE	PE00865884	19600912
HeNan Team s.n	PE	PE00865891	19590708
HengDuanShan Team 02005	PE	PE00864206	19810723
HengDuanShan Team 02005	PE	PE00864207	19810723
HengDuanShan Team 02005	PE	PE00865971	19810723
HengDuanShan Team 02005	PE	PE00865972	19810723
HengDuanShan Team 2467	PE	PE00864208	19810803
HengDuanShan Team 2467	PE	PE00865969	19810803
HengDuanShan Team 2467	PE	PE00865970	19810803
HengDuanShan Team 2467	PE	PE01982481	19810803
Hong-Fu Zhou & He-Li Su 109914	PE	PE00865926	19540804
HT Tsai 52019	PE	PE00866008	19320913
HT Tsai 52019	PE	PE00866009	19320913
HT Tsai 53724	PE	PE00866013	19330817
HT Tsai 53860	PE	PE00866014	19330727
JF Rock 18194	PE	PE00865756	19290900
JI Jeon et al. SI1299	PE	PE01900771	20100810
Jia-Rui Chen 92434A	PE	PE	19921001
Jia-Rui Chen 92449	PE	PE	19921001
Ji-Hua Xiong et al. 31889	PE	PE00865927	19520802
Ji-Hua Xiong et al. 32684	PE	PE00865928	19520907
KangZang Plant Research Team 10-2542	PE	PE01873305	20100821
KangZang Plant Research Team 10-2763	PE	PE01873315	20100825
Ke-Jian Guan et al. 1346	PE	PE00865768	19630803
Ke-Jian Guan et al. 1346	PE	PE00865917	19630803
Ke-Jian Guan et al. 1346	PE	PE00866041	19630803
KM Feng 2890	PE	PE00866040	19390831
KM Liou 5091	PE	PE00865889	19350812
KM Liou 5109	PE	PE00865888	19350814

KM Liou 5127	PE	PE00865890	19350814
KS Hao 4326	PE	PE00865897	19320917
Kun-Jun Fu 17668	PE	PE	19780727
Kun-Jun Fu 17668	PE	PE	19780727
Kun-Jun Fu 5903	PE	PE00865893	19520913
KunMing Work Station of Plant Research Institute 21289	PE	PE00866043	
KunMing Work Station of Plant Research Institute 22526	PE	PE00861992	19590806
linXing Group s.n	PE	PE00865919	19550000
Li-Song Wang & Jian-Fei Ye 228	PE	PE	20090828
Li-Song Wang & Jian-Fei Ye 228	PE	PE	20090828
Miao Liu et al. H10028	PE	PE01041313	20050626
Min-Zhi Qian 1408	PE	PE00865912	19580000
MK Feng 1621	PE	PE00866003	19390712
MK Feng 5573	PE	PE00866001	19400718
MK Feng 5892	PE	PE00866002	19400803
Ping-Hua Yu 786	PE	PE00865965	19570913
Ping-Hua Yu 963	PE	PE00865973	19570927
Plant Geography Investigation Team 603	PE	PE01374776	19780720
Plant Geography Investigation Team 603	PE	PE01374777	19780720
Qi-Guang Zhao 30441	PE	PE00866005	19390725
Qin-Er Yang 94138	PE	PE01869777	19990910
Qin-Er Yang 9606	PE	PE01869779	19960731
Qin-Er Yang et al. 180	PE	PE	19980829
Qin-Er Yang et al. 180	PE	PE	19980829
Qin-Er Yang et al. 180	PE	PE	19980829
Qin-Er Yang et al. 180	PE	PE	19980829
Qin-Er Yang et al. 180	PE	PE	19980829
Qin-Er Yang et al. 180	PE	PE	19980829
Qin-Er Yang et al. 180	PE	PE	19980829
Qin-Er Yang et al. 180	PE	PE	19980829
Qin-Er Yang et al. 452	PE	PE	19980921
Qin-Er Yang et al. 452	PE	PE	19980921
QinLin Team 10612	PE	PE	19580915
QinLin Team 10612	PE	PE	19580915
Qi-Wu Wang 63972	PE	PE00866024	19350600
Qi-Wu Wang 63972	PE	PE00866025	19350600
Qi-Wu Wang 64600	PE	PE00866022	19350700
Qi-Wu Wang 64600	PE	PE00866023	19350700
Qi-Wu Wang 67261	PE	PE00866016	19351000
Qi-Wu Wang 67261	PE	PE00866017	19351000

Qi-Wu Wang 68534	PE	PE00866020	19350800
Qi-Wu Wang 68534	PE	PE00866021	19350800
Qi-Wu Wang 68630	PE	PE00866026	19350800
Qi-Wu Wang 68630	PE	PE00866027	19350800
Qi-Wu Wang 68768	PE	PE00866018	19350800
Qi-Wu Wang 68768	PE	PE00866019	19350800
RC Ching 20994	PE	PE00866006	19390715
Ren-Chang Qin 23419	PE	PE00865998	19290929
Ren-Chang Qin 23419	PE	PE00865999	19290929
Ren-Chang Qin 24016	PE	PE00865996	19290823
Ren-Chang Qin 24016	PE	PE00865997	19290823
Ren-Chang Qin 24370	PE	PE00866000	19290906
Ren-Chang Qin 24370	PE	PE	19290906
RP Farges	PE	PE01899425	
Ru Long et al. 090807003	PE	PE01857430	20090807
Ru Long et al. 090807003	PE	PE01857431	20090807
Ru Long et al. 090807003	PE	PE01857432	20090807
Ru Long et al. 090807003	PE	PE01857433	20090807
Shen-E Liu 21278	PE	PE00865980	19461005
Sheng-Xiang Yu et al. 4603	PE	PE	20110730
Sheng-Xiang Yu et al. 4603	PE	PE	20110730
Sheng-Xiang Yu et al. 4875	PE	PE	20110803
Sheng-Xiang Yu et al. 4875	PE	PE	20110803
Shu-Zhen Gao 1212	PE	PE00865905	19550000
Su-Gong Wu 2138	PE	PE00865937	19590725
TaiBai Team 55242	PE	PE	19550712
TaiBai Team 55242	PE	PE	19550712
TH Tu 589	PE	PE00866140	19350813
Tibet Team 12215	PE	PE00864210	19830721
Tibet Team 12215	PE	PE00865918	19830721
Tibet Team 13052	PE	PE00864212	19830819
Tibet Team 13174	PE	PE00861989	19830821
Tibet Team 13174	PE	PE00861990	19830821
Tibet Team 2189	PE	PE00864202	19810706
Tibet Team 2189	PE	PE00864203	19810706
Tibet Team 2254	PE	PE00864201	19810709
Tibet Team 2254	PE	PE00864209	19810709
Tibet Team 2308	PE	PE00864204	19810710
Tibet Team 2308	PE	PE01982480	19810710
Tibet Team 2518	PE	PE00861991	19810709
Tibet Team 2518	PE	PE01982474	19810709
Tibet Team 8556	PE	PE00864205	19820725
Tibet Team 8556	PE	PE01982473	19820725
TN Liou & PC Tsoong 412	PE	PE00865899	19370829

TN Liou & PC Tsoong 412	PE	PE00865900	19370829
TN Liou & PC Tsoong 711	PE	PE00865898	19370908
TP Wang 1675	PE	PE00865901	19330801
TP Wang 1675	PE	PE00865902	19330801
TP Wang 1675	PE	PE00865903	19330801
TP Wang 1675	PE	PE00865904	19330801
Tsun-shen Ying 333	PE	PE	19870723
Tsun-shen Ying 333	PE	PE	19870723
Tsun-Shen Ying et al. 0333	PE	PE01536763	19870723
TT Yu 12079	PE	PE00866034	19370709
TT Yu 12079	PE	PE00866035	19370709
TT Yu 12079	PE	PE00866045	
TT Yu 12194	PE	PE00866032	19370716
TT Yu 12194	PE	PE00866033	19370716
TT Yu 12194	PE	PE00866054	
TT Yu 15397	PE	PE00866028	19370803
TT Yu 15397	PE	PE00866029	19370803
TT Yu 15397	PE	PE00866050	
TT Yu 16946	PE	PE00866010	19370724
TT Yu 16946	PE	PE00866011	19370724
TT Yu 16946	PE	PE00866012	19370724
TT Yu 16946	PE	PE00866047	
TT Yu 16946	PE	PE00866052	
TT Yu 22207	PE	PE00865984	19380806
TT Yu 22207	PE	PE00865985	19380806
TT Yu 22402	PE	PE00865986	19380818
TT Yu 22402	PE	PE00865987	19380818
TT Yu 22402	PE	PE00865989	19370818
TT Yu 22493	PE	PE00865990	19370826
TT Yu 22493	PE	PE00865991	19370826
TT Yu 22493	PE	PE00865992	19370826
TT Yu 22600	PE	PE00865993	19380831
TT Yu 22600	PE	PE00865994	19380831
TT Yu 22600	PE	PE00865995	19380831
TT Yu 22782	PE	PE00865982	19371008
TT Yu 22782	PE	PE00865983	19371008
TT Yu 22782	PE	PE00866015	19371008
TT Yu 3931	PE	PE00865939	19340831
TT Yu 3931	PE	PE00865957	19340831
TT Yu 6992	PE	PE00865946	19370711
TT Yu 6992	PE	PE00865947	19370711
TT Yu 6992	PE	PE00866046	
TT Yu 7226	PE	PE00865948	19370704
TT Yu 7226	PE	PE00865949	19370704

TT Yu 7603	PE	PE00865943	19370805
TT Yu 7603	PE	PE00865944	19370805
TT Yu 7603	PE	PE00865945	19370805
TT Yu 8894	PE	PE00866036	19370711
TT Yu 8894	PE	PE00866037	19370711
TT Yu 8894	PE	PE00866055	
TT Yu 8971	PE	PE00866038	19370714
TT Yu 8971	PE	PE00866039	19370714
TT Yu 8971	PE	PE00866048	
TT Yu 8971	PE	PE00866051	
TT Yu 9787	PE	PE00866030	19370823
TT Yu 9787	PE	PE00866031	19370823
TT Yu 9787	PE	PE00866049	
Unknown Collector	PE	PE01869774	19750908
Unknown Collector 0586	PE	PE00865922	19590609
Unknown Collector 0586	PE	PE00865923	19590609
Unknown Collector 10612	PE	PE01387860	19580915
Unknown Collector 10838	PE	PE00865936	19580717
Unknown Collector 11455	PE	PE00866057	19380828
Unknown Collector 13052	PE	PE00864211	19830819
Unknown Collector 135	PE	PE00865915	19730805
Unknown Collector 1568	PE	PE00865924	19590802
Unknown Collector 1568	PE	PE00865925	19590802
Unknown Collector 1853	PE	PE00865920	19580201
Unknown Collector 1853	PE	PE00865921	19580201
Unknown Collector 21124	PE	PE00866042	
Unknown Collector 34041	PE	PE01869783	19770813
Unknown Collector 34480	PE	PE00865886	19590708
Unknown Collector 34480	PE	PE00865887	19590708
Unknown Collector 35112	PE	PE00865885	19590826
Unknown Collector 3814	PE	PE00865932	19590711
Unknown Collector 3814	PE	PE00865938	19590711
Unknown Collector 3823	PE	PE00865933	19590712
Unknown Collector 4022	PE	PE00865934	19590719
Unknown Collector 5654	PE	PE00865913	
Unknown Collector 63836	PE	PE00865955	
Unknown Collector 822	PE	PE00865958	19230000
Unknown Collector 960381	PE	PE	19960712
Unknown Collector 960381	PE	PE	19960712
Unknown Collector 960383	PE	PE	19960712
Unknown Collector 960383	PE	PE	19960712
Unknown Collector 9796	PE	PE00865966	19600707
Unknown Collector s.n	PE	PE00866056	
Vegetation Research Team 12316	PE	PE00865935	19760719

Wei-Lie Chen et al. 8671	PE	PE00862149	19840728
Wen-Guang Hu & Zhu He 10426	PE	PE00865790	19510719
Wen-Guang Hu & Zhu He 10487	PE	PE00865791	19510724
Wen-Pei Fang 17597	PE	PE00865952	19410820
Wen-Pei Fang 2908	PE	PE00865940	19280813
Wen-Pei Fang 2908	PE	PE00866143	19280813
Wen-Pei Fang 3549	PE	PE00865942	19280925
Xiao-Hua Jin et al. DLJ-ET 1673	PE	PE02003642	20080708
Xiao-Hua Jin et al. SET-ET 45	PE	PE	20090812
Xiao-Hua Jin et al. ST2071	PE	PE01979365	20130805
Xiao-Hua Jin et al. ST2071	PE	PE01979366	20130805
Xiao-Hua Jin et al. YN-ET 1483	PE	PE	20090716
Xun-Lin Yu et al. 080026	PE	PE01869781	20080807
Xun-Lin Yu et al. 80026	PE	PE	20080807
Xun-Lin Yu et al. 80026	PE	PE	20080807
Y Tsiang 11510	PE	PE00866053	
Yao-Dong Chen et al. 2067	PE	PE00861987	19880930
Yao-Dong Chen et al. 2067	PE	PE00862148	19880930
You-Sheng Chen 4325	PE	PE	20020630
You-Sheng Chen 4325	PE	PE	20020630
Yunnan Tropical Biological Resources Comprehensive Inspection Team 2330	PE	PE00866044	
Zheng-Bo Feng et al. 4137	PE	PE01869780	20070727
Zheng-Yi Wu et al. 88	PE	PE01982475	19800825
Zhen-Shu Liu 822	PE	PE00865950	19340000
Zhi-Jian Yin et al. 1149	PE	PE01899016	20090713
Zhi-Jian Yin et al. 1265	PE	PE01898380	20090715
Zhi-Ying Zhang 16798	PE	PE00865907	
ZhongDian Team 1385	PE	PE00029360	19620826
ZhongDian Team 1385	PE	PE00865975	19620826
ZhongDian Team 1965	PE	PE00865976	19620909
ZhongDian Team 1965	PE	PE00865977	19620909
ZhongDian Team 850	PE	PE00865978	19620815
ZhongDian Team 850	PE	PE00865979	19620815
Zhong-Lun Wu 12277	PE	PE00865962	
Zhong-Lun Wu 12277	PE	PE00865964	
Zhu,Chen,Xu,Wang 1996	PE	PE01869776	19990910
Zi-Fu Song 39041	PE	PE00865953	19540000
Zuo-Bin Wang 15667	PE	PE00865892	19520708
Zuo-Bin Wang 16437	PE	PE00865894	19520903
BaiSha Team 452	SM	SM706501185	19790823
BaiSha Team 452	SM	SM	19790823
BeiChuan Team 382	SM	SM706501160	19780804

HongYa Group 1011	SM	SM706501182	19790816
HongYa Group 1011	SM	SM	19790816
LuShan Team 78-0819	SM	SM706501114	19780826
MuXian 578	SM	SM706501202	19780805
MuXian 578	SM	SM	19780805
NanChang 1071	SM	SM706501200	19790708
NanChang 1071	SM	SM	19790708
NingZu 348	SM	SM706501201	19780714
NingZu 348	SM	SM	19780714
TianJin General Investigation 78-562	SM	SM706501113	19780801
Unknown Collector 11889	SM	SM706501204	19610910
Unknown Collector 120	SM	SM706501207	19780701
Unknown Collector 120	SM	SM	19780701
Unknown Collector 120	SM	SM	19780701
Unknown Collector 120	SM	SM	19780701
Unknown Collector 1568	SM	SM706501194	19590802
Unknown Collector 1853	SM	SM706501195	19590821
Unknown Collector 20	SM	SM706501150	19780701
Unknown Collector 2138	SM	SM	
Unknown Collector 25044	SM	SM706501205	19600804
Unknown Collector 25044	SM	SM	19600804
Unknown Collector 25083	SM	SM706501203	19600807
Unknown Collector 25174	SM	SM706501206	19600814
Unknown Collector 288	SM	SM706501196	19790718
Unknown Collector 288	SM	SM	19790718
Unknown Collector 339	SM	SM	19780814
Unknown Collector 343	SM	SM706501199	19780702
Unknown Collector 343	SM	SM	19780702
Unknown Collector 343	SM	SM	19780702
Unknown Collector 414	SM	SM706501191	19790716
Unknown Collector 414	SM	SM	19790716
Unknown Collector 416	SM	SM706501192	19780809
Unknown Collector 416	SM	SM	19780809
Unknown Collector 4167	SM	SM706501188	19570820
Unknown Collector 4167	SM	SM	19570820
Unknown Collector 499	SM	SM706501193	19790629
Unknown Collector 499	SM	SM	19790629
Unknown Collector 644	SM	SM706501187	19780720
Unknown Collector 644	SM	SM	19780720
WanYuan Team 534	SM	SM706501186	19780801
WanYuan Team 534	SM	SM	19780801
YaAn Team 927	SM	SM706501183	19780813
YaAn Team 927	SM	SM706501184	19780813

Yuan Han 599	SM	SM706501198	19780819
ZhaoJueJiCha Team 655	SM	SM706501197	19790630
ZhaoJueJiCha Team 655	SM	SM	19790630
Gui-Ling Qu 3231	SZ	SZ00179815	19360723
Gui-Ling Qu 3524	SZ	SZ00179817	19360811
Guo-Mei Feng 21289	SZ	SZ00179893	19550901
Guo-Mei Feng 21631	SZ	SZ00180201	19551103
Ming-Yuan Fang 203	SZ	SZ00185050	19860705
Pin-Yi Mao 00600	SZ	SZ00180202	19561103
Qing-Sheng Zhao & Ya-Bin Yang 7323	SZ	SZ00179974	19780711
Qing-Sheng Zhao & Ya-Bin Yang 7323	SZ	SZ00179976	19780711
Qing-Sheng Zhao et al. 6736	SZ	SZ00179824	19780723
Qing-Sheng Zhao et al. 6736	SZ	SZ00179970	19780723
Qing-Sheng Zhao et al. 6736	SZ	SZ00179972	19780723
Qing-Sheng Zhao et al. 6840	SZ	SZ00179831	19780728
Qing-Sheng Zhao et al. 6840	SZ	SZ00179969	19780728
Qing-Sheng Zhao et al. 6840	SZ	SZ00179971	19780728
Qing-Sheng Zhao et al. 6840	SZ	SZ00179973	19780728
Qing-Sheng Zhao et al. 7885	SZ	SZ00179827	19780731
Qing-Sheng Zhao et al. 7885	SZ	SZ00179821	19780731
T.K.Wang 0605	SZ	SZ00179968	19380800
Tong-Pei Yi 004	SZ	SZ00184779	19990919
Tong-Pei Yi 004	SZ	SZ00184786	19990919
Tong-Pei Yi 734	SZ	SZ00184870	19990920
Unknown Collector	SZ	SZ00179856	
Unknown Collector	SZ	SZ00179860	
Unknown Collector 0019	SZ	SZ00185049	19930703
Unknown Collector 189	SZ	SZ00185051	
Unknown Collector 79077	SZ	SZ00179975	19790718
Unknown Collector 99113	SZ	SZ00184752	19990731
Wen-Guang Hu & Zhu He 10426	SZ	SZ00179828	19510719
Wen-Guang Hu & Zhu He 10426	SZ	SZ00179829	19510719
Wen-Guang Hu & Zhu He 10487	SZ	SZ00179822	19510724
Wen-Guang Hu & Zhu He 10487	SZ	SZ00179825	19510724
Wen-Guang Hu & Zhu He 11130	SZ	SZ00179826	19510906
Wen-Pei Fang & Wen-Guang Hu 51-533	SZ	SZ00179869	19540716
Wen-Pei Fang 51-848	SZ	SZ00179867	19540730
Xing-Lin Jiang 10832	SZ	SZ00179818	19580717
Xing-Lin Jiang 10838	SZ	SZ00179820	19580717
Xing-Lin Jiang 36411	SZ	SZ00179816	19530723
Xi-Tao Cai 53724	SZ	SZ00179830	19330817

Xi-Tao Cai 53860	SZ	SZ00249779	19330727
Xiu-Ying Hu 1343	SZ	SZ00179966	19390708
Ya-Bin Yang 7323	SZ	SZ00179832	19780711
Ya-Bin Yang 7323	SZ	SZ00179977	19780711
Ying Jiang 11510	SZ	SZ00179823	19330400
Zhen-Ju Zhao et al. 114897	SZ	SZ00180129	19810711
Zhen-Ju Zhao et al. 115239	SZ	SZ00180128	19810817
Zhen-Shu Liu 822	SZ	SZ00179814	
Zhong-Ming Tan & Shu-Hua Yu 123124	SZ	SZ00180143	19870810
Zhong-Ming Tan & Shu-Hua Yu 123124	SZ	SZ00180144	19870810
Zhong-Ming Tan & Shu-Hua Yu 123124	SZ	SZ00180145	19870810
Zhong-Ming Tan & Shu-Hua Yu 123125	SZ	SZ00180233	19870810
Zhong-Ming Tan & Shu-Hua Yu 123125	SZ	SZ00180234	19870810
Zhong-Ming Tan & Shu-Hua Yu 123125	SZ	SZ00180237	19870810
Zhong-Ming Tan & Shu-Hua Yu 123128	SZ	SZ00180140	19870810
Zhong-Ming Tan & Shu-Hua Yu 123128	SZ	SZ00180255	19870810
Zhong-Ming Tan & Shu-Hua Yu 123128	SZ	SZ00180256	19870810
Zhong-Ming Tan & Shu-Hua Yu 123129	SZ	SZ00180146	19870810
Zhong-Ming Tan & Shu-Hua Yu 123129	SZ	SZ00180152	19870810
Zhong-Ming Tan & Shu-Hua Yu 123129	SZ	SZ00180161	19870810
Zhong-Ming Tan & Shu-Hua Yu 123130	SZ	SZ00180147	19870810
Zhong-Ming Tan & Shu-Hua Yu 123130	SZ	SZ00180148	19870810
Zhong-Ming Tan & Shu-Hua Yu 123130	SZ	SZ00180151	19870810
Zhong-Ming Tan & Shu-Hua Yu 123141	SZ	SZ00180250	19870810
Zhong-Ming Tan & Shu-Hua Yu 123141	SZ	SZ00180251	19870810
Zhong-Ming Tan & Shu-Hua Yu 123141	SZ	SZ00180253	19870810

Zhong-Ming Tan & Shu-Hua Yu 123147	SZ	SZ00180248	19870810
Zhong-Ming Tan & Shu-Hua Yu 123147	SZ	SZ00180249	19870810
Zhong-Ming Tan & Shu-Hua Yu 123147	SZ	SZ00180252	19870810
Zhong-Ming Tan & Shu-Hua Yu 123199	SZ	SZ00180142	19870811
Zhong-Ming Tan & Shu-Hua Yu 123199	SZ	SZ00180149	19870811
Zhong-Ming Tan & Shu-Hua Yu 123199	SZ	SZ00180150	19870811
Zhong-Ming Tan & Shu-Hua Yu 123213	SZ	SZ00180125	19870811
Zhong-Ming Tan & Shu-Hua Yu 123213	SZ	SZ00180205	19870811
Zhong-Ming Tan & Shu-Hua Yu 123213	SZ	SZ00180206	19870811
Zhong-Ming Tan & Shu-Hua Yu 87010	SZ	SZ00180228	19870829
Zhong-Ming Tan & Shu-Hua Yu 87010	SZ	SZ00180229	19870829
Zhong-Ming Tan & Shu-Hua Yu 87010	SZ	SZ00180242	19870829
Zhong-Ming Tan & Shu-Hua Yu 87025	SZ	SZ00180137	19870829
Zhong-Ming Tan & Shu-Hua Yu 87025	SZ	SZ00180225	19870829
Zhong-Ming Tan & Shu-Hua Yu 87025	SZ	SZ00180227	19870829
Zhong-Ming Tan & Shu-Hua Yu 87031	SZ	SZ00180226	19870829
Zhong-Ming Tan & Shu-Hua Yu 87048	SZ	SZ00180138	19870831
Zhong-Ming Tan & Shu-Hua Yu 87048	SZ	SZ00180139	19870831
Zhong-Ming Tan & Shu-Hua Yu 87048	SZ	SZ00180214	19870831
Zhong-Ming Tan & Shu-Hua Yu 87052	SZ	SZ00180212	19870831
Zhong-Ming Tan & Shu-Hua Yu 87052	SZ	SZ00431950	19870831
Zhong-Ming Tan & Shu-Hua Yu 87052	SZ	SZ00180213	19870831

Zhong-Ming Tan & Shu-Hua Yu 87054	SZ	SZ00180210	19870831
Zhong-Ming Tan & Shu-Hua Yu 87054	SZ	SZ00180240	19870831
Zhong-Ming Tan & Shu-Hua Yu 87054	SZ	SZ00180241	19870831
Zhong-Ming Tan & Shu-Hua Yu 87055	SZ	SZ00180211	19870831
Zhong-Ming Tan & Shu-Hua Yu 87055	SZ	SZ00180243	19870831
Zi-Fu Song	SZ	SZ00179863	19540000
Zi-Fu Song 39041	SZ	SZ00179819	19540714
Zi-Fu Song 414068	SZ	SZ00179967	19540000
Chuan-Fen Wu 79-060	WCNU	WCNU0004750	19790708
Chuan-Fen Wu 79-060	WCNU	WCNU0004748	19790708
Shi-Xue Zhang 79-237	WCNU	WCNU0004745	19790707
Third Group 555	WCNU	WCNU0004751	19790716
Unknown Collector 001041	WCNU	WCNU0021102	19790803
Unknown Collector 002709	WCNU	WCNU0020223	19800815
Unknown Collector 002709	WCNU	WCNU0020224	19800815
Unknown Collector 002709	WCNU	WCNU0020225	19800815
Unknown Collector 00433	WCNU	WCNU0004756	19780806
Unknown Collector 00433	WCNU	WCNU0004755	19780806
Unknown Collector 2167	WCNU	WCNU0004746	19770719
Unknown Collector 2579	WCNU	WCNU	19730820
Unknown Collector 2579	WCNU	WCNU0004757	19730820
Unknown Collector 3151	WCNU	WCNU	19800815
Unknown Collector 79-396	WCNU	WCNU0027555	19790718
Unknown Collector 79-396	WCNU	WCNU0027556	19790718
Unknown Collector 79-396	WCNU	WCNU0027557	19790718
Unknown Collector wl-3101	WCNU	WCNU00015877	20160905
Unknown Collector wl-3101	WCNU	WCNU00015879	20160905
Unknown Collector wl-3101	WCNU	WCNU00015876	20160905
Unknown Collector wl-3101	WCNU	WCNU00015880	20160905
Unknown Collector wl-3101	WCNU	WCNU00015881	20160905
Unknown Collector wl-3101	WCNU	WCNU00015882	20160905
Xun-Sun Yu 06451	WCNU	WCNU0004747	19740705
ZhengHe Research Team 003709	WCNU	WCNU0020222	19800815
Parnassia delavayi			
Ce Shang I"-222	BJFC	BJFC	20130726
Jin-Yu Li & Jin Zhang SN201107042	BJFC	BJFC	20110812
Lei Xie EM07	BJFC	BJFC	20150901
Lei Xie EM29	BJFC	BJFC	20150901

Li He & Jun Zhao HL36	BJFC	BJFC	20160714
Li He & Xing-Xing Mao PH20120614-05	BJFC	BJFC	20120614
Li He et al. PH20120625-11	BJFC	BJFC	20120625
Li He et al. PH20120716-02	BJFC	BJFC	20120716
Li He et al. PH20120802-16	BJFC	BJFC	20120802
Xing-Xing Mao 2013072923	BJFC	BJFC	20130729
Yu-Min Shu & Feng-Bin Zhao sz003	BJFC	BJFC	20140729
Yu-Min Shu & Feng-Bin Zhao sz006	BJFC	BJFC	20140729
Yu-Min Shu & Feng-Bin Zhao sz028	BJFC	BJFC	20140729
Yu-Min Shu & Feng-Bin Zhao sz030	BJFC	BJFC	20140729
Yu-Min Shu & Rong-Yan Deng sd703	BJFC	BJFC	20151013
Yu-Ming Shu & Lei Wang sw361	BJFC	BJFC	20150801
Yu-Ming Shu & Lei Wang sw364	BJFC	BJFC	20150801
Yu-Ming Shu & Lei Wang sw367	BJFC	BJFC	20150801
Yu-Ming Shu & Lei Wang sw372	BJFC	BJFC	20150801
Yu-Ming Shu & Lei Wang sw414	BJFC	BJFC	20150803
Yu-Ming Shu & Lei Wang sw417	BJFC	BJFC	20150803
Yu-Ming Shu & Lei Wang sw418	BJFC	BJFC	20150803
Yu-Ming Shu & Lei Wang sw423	BJFC	BJFC	20150803
Yu-Ming Shu & Lei Wang sw437	BJFC	BJFC	20150805
Yu-Ming Shu & Lei Wang sw438	BJFC	BJFC	20150805
Yu-Ming Shu & Lei Wang sw439	BJFC	BJFC	20150805
Yu-Ming Shu & Lei Wang sw497	BJFC	BJFC	20150812
Yu-Ming Shu & Lei Wang sw498	BJFC	BJFC	20150812
Yu-Ming Shu & Lei Wang sw527	BJFC	BJFC	20150816
Guang-Hui Yang 59726	CDBI	CDBI0039573	19581009
Qing-Sheng Zhao et al. 6736	CDBI	CDBI0039161	19780723
Qing-Sheng Zhao et al. 6736	CDBI	CDBI0039162	19780723
Qing-Sheng Zhao et al. 6736	CDBI	CDBI0039163	19780723
Qing-Sheng Zhao et al. 6840	CDBI	CDBI0039727	19780728
Qing-Sheng Zhao et al. 6840	CDBI	CDBI0038364	19780728
Qing-Sheng Zhao et al. 6840	CDBI	CDBI0038365	19780728
Qing-Sheng Zhao et al. 7885	CDBI	CDBI0038500	19780731
Qing-Sheng Zhao et al. 7885	CDBI	CDBI0038501	19780731
Qing-Sheng Zhao et al. 7885	CDBI	CDBI0039155	19780731
Qing-Sheng Zhao et al. 7885	CDBI	CDBI0039156	19780731
Tai-Chang 20612	CDBI	CDBI0038594	19790717
Tibet Team 2189	CDBI	CDBI0038741	19810706

Tibet Team 2189	CDBI	CDBI0039420	19810706
Tibet Team 2254	CDBI	CDBI0039307	19810709
Tibet Team 2254	CDBI	CDBI0038431	19810709
Tibet Team 2308	CDBI	CDBI0039185	19810710
Tibet Team 2308	CDBI	CDBI0039431	19810710
Tibet Team 2518	CDBI	CDBI0039088	19810709
Tibet Team 2518	CDBI	CDBI0039396	19810709
Unknown Collector 1568	CDBI	CDBI0039056	19590802
Unknown Collector 1568	CDBI	CDBI0039561	19590802
Unknown Collector 4778	CDBI	CDBI0039723	19740704
Unknown Collector 4778	CDBI	CDBI0039724	19740704
Unknown Collector 4778	CDBI	CDBI0039569	19740704
Vegetation Group 31397	CDBI	CDBI0037155	19830820
Vegetation Group 31397	CDBI	CDBI0037156	19830820
Vegetation Group 31397	CDBI	CDBI0037157	19830820
Ya-Bin Yang 7323	CDBI	CDBI0039582	19780711
Ya-Bin Yang 7323	CDBI	CDBI0039583	19780711
Ya-Bin Yang 7323	CDBI	CDBI0039154	19780711
Yu-Rong Xu 23439	CDBI	CDBI0037158	19800911
Yu-Rong Xu 23439	CDBI	CDBI0037151	19800911
Ze-Rong Zhang et al. 124	CDBI	CDBI0037159	19630814
Ze-Rong Zhang et al. 124	CDBI	CDBI0037160	19630814
Zhen-Ju Zhao et al. 114897	CDBI	CDBI0039595	19810711
Zhen-Ju Zhao et al. 115239	CDBI	CDBI0039594	19810817
Zheng-Yu Liu 782625	IMC	IMC000014623	19780812
Zheng-Yu Liu 782625	IMC	IMC000014624	19780812
Zheng-Yu Liu 782625	IMC	IMC000014625	19780812
Zheng-Yu Liu 782625	IMC	IMC000014658	19780812
Ai-Hua Li 000157	KUN	KUN0768620	20060915
Cai-Qi Li 3242	KUN	KUN0437203	19400810
Cai-Qi Li 3954	KUN	KUN0437207	19400810
China & Germany Team 88-064	KUN	KUN0437115	19880724
Chong-Yun Zhao 20804	KUN	KUN0437108	19390723
Chong-Yun Zhao 20804	KUN	KUN0437109	19390723
Chong-Yun Zhao 20804	KUN	KUN0437110	19390723
Cong-Li Yang 75-15	KUN	KUN0437127	19750909
De-Ding Tao et al. 1002	KUN	KUN0437111	19820908
De-Jun Yu 12079	KUN	KUN0437078	19370709
De-Jun Yu 12194	KUN	KUN0437073	19370716
De-Jun Yu 12194	KUN	KUN0437072	19370716
De-Jun Yu 15397	KUN	KUN0437074	19370803
De-Jun Yu 15397	KUN	KUN0437075	19370803
De-Jun Yu 16946	KUN	KUN0437088	19380724
De-Jun Yu 16946	KUN	KUN0437089	19380724

De-Jun Yu 22207	KUN	KUN0437090	19380806
De-Jun Yu 22207	KUN	KUN0437091	19380806
De-Jun Yu 22402	KUN	KUN0437079	19380818
De-Jun Yu 22493	KUN	KUN0437082	19380826
De-Jun Yu 22493	KUN	KUN0437083	19380826
De-Jun Yu 22600	KUN	KUN0437080	19380831
De-Jun Yu 22600	KUN	KUN0437081	19380831
De-Jun Yu 22782	KUN	KUN0437084	19381008
De-Jun Yu 22782	KUN	KUN0437085	19381008
De-Jun Yu 6992	KUN	KUN0437189	19370711
De-Jun Yu 7603	KUN	KUN0437190	19370805
De-Jun Yu 8894	KUN	KUN0437093	19370711
De-Jun Yu 8971	KUN	KUN0437092	19370714
De-Jun Yu 8971	KUN	KUN0437094	19370714
De-Jun Yu 9787	KUN	KUN0437076	19370823
De-Jun Yu 9787	KUN	KUN0437077	19370823
Department of biology, Yunnan University 229	KUN	KUN0437157	19560705
Dian North-East Group 407	KUN	KUN0437130	19640726
Dian North-East Group 407	KUN	KUN0437133	19640726
Dian North-East Group 511	KUN	KUN0437131	19640813
Dian North-East Group 511	KUN	KUN0437132	19640813
Dian North-West JinSha River Team 63-6124	KUN	KUN0437134	19630725
Dian North-west JinSha River Team 63-6124	KUN	KUN0437135	19630725
Ding Wu & Jin-Mei Lu 3074	KUN-1	KUN-1	20030724
Ding WU & Shu-Dong Zhang 5011	KUN-1	KUN-1	20050814
Ding Wu et al. 2004	KUN-1	KUN-1	20020826
DuLongJiang Research Team 7041	KUN	KUN0437095	19910522
DuLongJiang Research Team 7041	KUN	KUN0437096	19910522
En-De Liu 5057	KUN	KUN0491260	20020712
En-De Liu 5057	KUN	KUN0491268	20020712
En-De Liu 6278	KUN	KUN0491207	20020813
En-De Liu 6278	KUN	KUN0491258	20020813
En-De Liu 6301	KUN	KUN0491257	20020813
En-De Liu et al. 2193	KUN	KUN0774512	20090806
Fan-Jin Dai 5247	KUN	KUN0437191	19390927
Fa-Zuan Wang 23402	KUN	KUN0437188	19310719
Guang-Hui Yang 59726	KUN	KUN0437208	19581009
Guo-Feng Li 63377	KUN	KUN0437209	19570000

Guo-Feng Li 83836	KUN	KUN0437210	19570000
Guo-Mei Feng 1621	KUN	KUN0437170	19390712
Guo-Mei Feng 1621	KUN	KUN0437172	19390712
Guo-Mei Feng 2081	KUN	KUN0437171	19390820
Guo-Mei Feng 2081	KUN	KUN0437173	19390820
Guo-Mei Feng 21289	KUN	KUN0437174	19550901
Guo-Mei Feng 21289	KUN	KUN0437175	19550901
Guo-Mei Feng 22526	KUN	KUN0437176	19590806
Guo-Mei Feng 22526	KUN	KUN0437177	19590806
Guo-Mei Feng 22526	KUN	KUN0437178	19590806
Guo-Mei Feng 2890	KUN	KUN0437204	19390831
Guo-Mei Feng 2890	KUN	KUN0437206	19390831
Guo-Mei Feng 5573	KUN	KUN0437166	19400718
Guo-Mei Feng 5573	KUN	KUN0437168	19400718
Guo-Mei Feng 5892	KUN	KUN0437167	19400803
Guo-Mei Feng 5892	KUN	KUN0437169	19400803
H Li 0433	KUN	KUN0137238	20020816
H Li 0433	KUN	KUN1307591	20020816
Hong Wang et al. 32088	KUN-1	KUN-1	20020826
Hong Wang et al. 894	KUN	KUN0679518	20030817
Hua Peng et al. 5024	KUN	KUN0137479	20030826
Hua Peng et al. 5024	KUN	KUN0137480	20030826
Hua Peng et al. 8516	KUN	KUN0773443	20080728
Hua Peng et al. 8516	KUN	KUN0773444	20080728
Hua Peng et al. 8870	KUN	KUN0773441	20080730
Hua Peng et al. 8870	KUN	KUN0773442	20080730
Jin-Mei Lu 3054	KUN-1	KUN-1	20030727
Ji-Pei Yue 3084	KUN-1	KUN-1	20030826
JM Delavay	KUN	KUN1217507	18830724
Kun-Jun Fu 8337	KUN	KUN0437186	19560731
LianDa 11455	KUN	KUN0437086	19380827
LianDa 21124	KUN	KUN0437087	
Lian-MIng Gao & Jin-Mei Lu 2317	KUN-1	KUN-1	20030819
Lian-Ming Gao 103061	KUN	KUN1242202	20100824
Lin-Han Liu 010601	KUN	KUN0437181	19640802
M Iabbe Delavay	KUN	KUN1221634	18900719
Na-Na Lin & Wen-Bin Yu 1025	KUN-1	KUN-1	20050708
NanShuiBeiDiao Team 9796	KUN	KUN0437153	19600707
NanShuiBeiDiao Team 9796	KUN	KUN0437154	19600707
PanGong Team 2084	KUN-1	KUN-1	20020912
Pei-Yuan Li 8589	KUN	KUN0437187	19560730
Ping-Hua Yu 786	KUN	KUN0437179	19570913
Ping-Hua Yu 786	KUN	KUN0437180	19570913

Ping-Hua Yu 963	KUN	KUN0437155	19570927
Ping-Hua Yu 963	KUN	KUN0437156	19570927
Qin Lin 791988	KUN	KUN0437123	19790801
Qin Lin 791988	KUN	KUN0437124	19790801
Qin Lin et al. 771689	KUN	KUN0437241	1985004
Qin Lin et al. 771689	KUN	KUN0437242	1985004
Qi-Wu Wang 64600	KUN	KUN0437145	19350700
Qi-Wu Wang 68534	KUN	KUN0437146	19350800
Qi-Wu Wang 68630	KUN	KUN0437147	19350800
Qi-Wu Wang 68768	KUN	KUN0437148	19350800
Ren-Chang Qin 20994	KUN	KUN0437104	19390715
Ren-Chang Qin 20994	KUN	KUN0437105	19390715
Ren-Chang Qin 21187	KUN	KUN0437106	19380731
Ren-Chang Qin 21187	KUN	KUN0437107	19380731
Ren-Chang Qin 23419	KUN	KUN0437101	19290729
Ren-Chang Qin 23419	KUN	KUN0437102	19290729
Ren-Chang Qin 24016	KUN	KUN0437103	19290823
Ren-Chang Qin 24370	KUN	KUN0437099	19290906
Ren-Chang Qin 24370	KUN	KUN0437100	19290906
Ren-Chang Qin 30441	KUN	KUN0437097	19390725
Ren-Chang Qin 30441	KUN	KUN0437098	19390725
RP Farges 29	KUN	KUN1230258	
RP Maire	KUN	KUN1225519	19210810
RP Maire	KUN	KUN1230234	19140815
Sheng-Tang Li 80-283	KUN	KUN0437125	19800726
Sheng-Tang Li 80-283	KUN	KUN0437126	19800726
Sino-US Joint Western Hubei Plant research Team 37	KUN	KUN0437182	19800824
Sino-US Joint Western Hubei Plant research Team 37	KUN	KUN0437183	19800824
Tibet Team 13052	KUN	KUN0437192	19830819
Tibet Team 13052	KUN	KUN0437194	19830819
Tibet Team 13174	KUN	KUN0437193	19830821
Tibet Team 13174	KUN	KUN0437195	19830821
Tibet Team 2189	KUN	KUN0437112	19810706
Tibet Team 2189	KUN	KUN0437120	19810707
Tibet Team 2254	KUN	KUN0437121	19810709
Tibet Team 2254	KUN	KUN0437122	19810709
Tibet Team 2308	KUN	KUN0437116	19810710
Tibet Team 2308	KUN	KUN0437117	19810710
Tibet Team 2518	KUN	KUN0437118	19810709
Tibet Team 2518	KUN	KUN0437119	19810709
Tibet Team 8556	KUN	KUN0437136	19820725
Tibet Team 8556	KUN	KUN0437137	19820725

Unknown Collector	KUN	KUN0437198	19820903
Unknown Collector	KUN	KUN0437212	
Unknown Collector 1212	KUN	KUN0437211	
Unknown Collector 1568	KUN	KUN0437200	19590802
Unknown Collector 1853	KUN	KUN0437199	19590821
Unknown Collector 2138	KUN	KUN0437202	
Unknown Collector 3823	KUN	KUN0437201	19590712
Unknown Collector 489	KUN	KUN0437138	
Wen-Cai Wang 100	KUN	KUN0437151	19580902
Wen-Cai Wang 100	KUN	KUN0437152	19580902
Xi-Tao Cai 52019	KUN	KUN0437141	19320913
Xi-Tao Cai 52019	KUN	KUN0437143	19320913
Xi-Tao Cai 53724	KUN	KUN0437142	19330817
Xi-Tao Cai 53724	KUN	KUN0437144	19330817
Xi-Tao Cai 53860	KUN	KUN0437139	19330727
Xi-Tao Cai 53860	KUN	KUN0437140	19330727
Ying Jiang 11510	KUN	KUN0437149	19330400
Ying Jiang 11510	KUN	KUN0437150	19330400
Ying Jiang 11510	KUN	KUN0437449	19330000
ZD Fang 990	KUN	KUN0437114	19880717
Zheng-Yi Wu 4279	KUN	KUN0437128	19760808
Zheng-Yi Wu 4279	KUN	KUN0437129	19760808
Zheng-Yi Wu et al. 86-289	KUN	KUN0437213	19860923
Zheng-Yi Wu et al. 88	KUN	KUN0437196	19800825
Zheng-Yi Wu et al. 88	KUN	KUN0437197	19800825
Zhi-Dan Wei et al. 5735	KUN	KUN1342383	20170801
Zhi-Jian Yin et al. 1265	KUN	KUN1215329	20090715
Zhi-Jian Yin et al. 1265	KUN	KUN1215479	20090715
ZhongDian Team 1385	KUN	KUN0437159	19620826
ZhongDian Team 1385	KUN	KUN0437161	19620826
ZhongDian Team 1965	KUN	KUN0437162	19620909
ZhongDian Team 1965	KUN	KUN0437163	19620909
ZhongDian Team 63-3415	KUN	KUN0437164	19630714
ZhongDian Team 63-3415	KUN	KUN0437165	19630714
ZhongDian Team 850	KUN	KUN0437158	19620816
ZhongDian Team 850	KUN	KUN0437160	19620816
Zuo-Bin Wang 15667	KUN	KUN0437185	19520708
Zuo-Bin Wang 16437	KUN	KUN0437184	19520903
A Monbeig	P	P03331424	1908
Delavay	P	P05556320	18890914
Delavay	P	P05556321	18890914
Delavay	P	P05583619	18890719
Delavay	P	P05583623	
Delavay	P	P06392627	18880729

Delavay	P	P06392628	18880729
Delavay	P	P06392629	18880729
Delavay	P	P06392633	18890904
Delavay	P	P06392635	18850911
Delavay	P	P06392668	18840901
Delavay 1	P	P05556380	18830724
Delavay 1	P	P05556385	18830724
Delavay 1	P	P06392626	18830724
Delavay 1	P	P06392632	
Delavay 130	P	P06392623	
Delavay 2	P	P06392622	18920700
Delavay 217	P	P05556394	18830816
Delavay 217	P	P05556395	18830816
Delavay 217	P	P06392624	
Delavay 72	P	P05494801	18840823
Delavay 72	P	P06392630	18840823
Delavay 72	P	P06392634	18840823
EH Wilson 4089	P	P05583613	
EH Wilson 4895	P	P03331598	19030000
Farges	P	P05556384	
Farges	P	P05556389	
Farges	P	P05583580	
Farges	P	P05583581	
Farges 27	P	P05494800	
Farges 27	P	P05556393	
Farges 27	P	P06392655	
Farges 27	P	P06392661	
Farges 27	P	P06392662	
Farges 27	P	P06392646	
Forrest 6196	P	P06392657	19100700
Fr Ducloux 4315	P	P03371245	19060800
Fr Ducloux 4315	P	P03371246	19060800
FT Wang	P	P03370640	1931
Henry 6542	P	P06392636	18890000
Henry 6542	P	P06392641	18890000
Henry 7260	P	P06392642	18890000
JM Delavay 130	P	P00709357	1883-8-7
JM Delavay 130	P	P00709358	1883-8-7
JM Delavay 217	P	P00709355	1883-8-16
JM Delavay 217	P	P00709356	1883-8-16
M Fr Ducloux 6407	P	P03331419	190907
Maire	P	P04446728	
Maire	P	P05608135	
McLaren 79	P	P06392639	19380000

Monbeig	P	P06865506	
Rock 16949	P	P06392638	19280800
Rock 18500	P	P06392650	19290700
Rock 5796	P	P06392648	19220000
Soulie	P	P06392640	
Soulie 13	P	P06392645	
Soulie 1304	P	P06865510	
Soulie 304	P	P06392647	
Soulie 304	P	P06392644	
Soulie 793	P	P05494769	
Soulie 818	P	P05608125	18930000
Soulie 818	P	P06392659	1893
Soulie 818	P	P06392660	1893
Unknown Collector	P	P06392658	
Unknown Collector 27	P	P06392654	
Wen-Pei Fang 2785	P	P06392651	19280811
Aug Henry 7260	PE	PE01982470	
BaShan Collection Team 2286	PE	PE01857424	20080725
BaShan Collection Team 2499	PE	PE01857425	20080726
Ben-Zhao Guo 899	PE	PE00865896	19510901
Cai-Fei Zhang 1061	PE	PE	20080727
Cai-Fei Zhang 1061	PE	PE	20080727
Cong-Rong Wang 1005	PE	PE01869782	19930713
CS Liu 822	PE	PE00865941	19340723
Da-Hua Du 589	PE	PE00865956	19350813
De-Jun Yu 3931	PE	PE00865951	19340831
Delavay	PE	PE	18830724
Dian North-west JinSha River Group 63-6124	PE	PE00865967	19630725
Dian North-west JinSha River Group 63-6124	PE	PE00865968	19630725
Dr Aug Henry 6542	PE	PE00865914	
Fa-Zuan Wang et al. 086	PE	PE00865895	19551003
Fa-Zuan Wang et al. 144	PE	PE00861986	19550929
feng 2081	PE	PE01982466	19390820
FT Wang 23402	PE	PE00865988	19310722
FT Wang 23402	PE	PE00866144	19310722
Fu-Ming Zhao 21187	PE	PE00866004	19390731
G Forrest 12487	PE	PE00866060	19140000
G Forrest 28537	PE	PE00866059	19300000
G Forrest 29147	PE	PE00866062	19300000
G Forrest 6196	PE	PE00866058	19100000
G.Forrest 28086	PE	PE00866061	19290400
General Investigation 14523	PE	PE00866730	19590000

General Investigation Specimen 20346	PE	PE00865881	19590000
General Investigation Specimen 20346	PE	PE00865882	19590000
Guang-Hui Yang 56455	PE	PE00865931	19590806
Guang-Hui Yang 56455	PE	PE00865954	19590806
Guang-Hui Yang 56633	PE	PE00866141	19570813
Guang-Hui Yang 57412	PE	PE00865930	19570926
Guang-Hui Yang 58765	PE	PE00861988	19580714
Guang-Hui Yang 58765	PE	PE00865916	19580714
Guang-Hui Yang 59728	PE	PE00865908	19581009
Guang-Yu Hong 4063	PE	PE02059342	10200713
Guang-Zhao Li 15542	PE	PE00862032	19970725
Gui-Ling Qu 3524	PE	PE00865959	19360000
Gui-Ling Qu 3524	PE	PE00865961	19360000
Gui-Ling Qu 3524	PE	PE00865963	19360000
Guo-Feng Li 63877	PE	PE00866142	19570829
Guo-Mei Feng 21289	PE	PE00865974	19550901
Guo-Xun Fu & Zhi-Song Zhang 1512	PE	PE00865909	19570903
Guo-Xun Fu & Zhi-Song Zhang 1512	PE	PE00865910	19570903
Guo-Xun Fu & Zhi-Song Zhang 1512	PE	PE00865911	19570903
Han-Chen Wang 1206	PE	PE00865981	19410800
Han-Chen Wang 4497	PE	PE00866007	19440813
HeNan Team 1233	PE	PE00865883	19600718
HeNan Team 2529	PE	PE00865884	19600912
HeNan Team s.n	PE	PE00865891	19590708
HengDuanShan Team 02005	PE	PE00864206	19810723
HengDuanShan Team 02005	PE	PE00864207	19810723
HengDuanShan Team 02005	PE	PE00865971	19810723
HengDuanShan Team 02005	PE	PE00865972	19810723
HengDuanShan Team 2467	PE	PE00864208	19810803
HengDuanShan Team 2467	PE	PE00865969	19810803
HengDuanShan Team 2467	PE	PE00865970	19810803
HengDuanShan Team 2467	PE	PE01982481	19810803
Hong-Fu Zhou & He-Li Su 109914	PE	PE00865926	19540804
HT Tsai 52019	PE	PE00866008	19320913
HT Tsai 52019	PE	PE00866009	19320913
HT Tsai 53724	PE	PE00866013	19330817
HT Tsai 53860	PE	PE00866014	19330727
JF Rock 18194	PE	PE00865756	19290900

JI Jeon et al. SI1299	PE	PE01900771	20100810
Jia-Rui Chen 92434A	PE	PE	19921001
Jia-Rui Chen 92449	PE	PE	19921001
Ji-Hua Xiong et al. 31889	PE	PE00865927	19520802
Ji-Hua Xiong et al. 32684	PE	PE00865928	19520907
KangZang Plant Research Team 10-2542	PE	PE01873305	20100821
KangZang Plant Research Team 10-2763	PE	PE01873315	20100825
Ke-Jian Guan et al. 1346	PE	PE00865768	19630803
Ke-Jian Guan et al. 1346	PE	PE00865917	19630803
Ke-Jian Guan et al. 1346	PE	PE00866041	19630803
KM Feng 2890	PE	PE00866040	19390831
KM Liou 5091	PE	PE00865889	19350812
KM Liou 5109	PE	PE00865888	19350814
KM Liou 5127	PE	PE00865890	19350814
KS Hao 4326	PE	PE00865897	19320917
Kun-Jun Fu 17668	PE	PE	19780727
Kun-Jun Fu 17668	PE	PE	19780727
Kun-Jun Fu 5903	PE	PE00865893	19520913
KunMing Work Station of Plant Research Institute 21289	PE	PE00866043	
KunMing Work Station of Plant Research Institute 22526	PE	PE00861992	19590806
linXing Group s.n	PE	PE00865919	19550000
Li-Song Wang & Jian-Fei Ye 228	PE	PE	20090828
Li-Song Wang & Jian-Fei Ye 228	PE	PE	20090828
Miao Liu et al. H10028	PE	PE01041313	20050626
Min-Zhi Qian 1408	PE	PE00865912	19580000
MK Feng 1621	PE	PE00866003	19390712
MK Feng 5573	PE	PE00866001	19400718
MK Feng 5892	PE	PE00866002	19400803
Ping-Hua Yu 786	PE	PE00865965	19570913
Ping-Hua Yu 963	PE	PE00865973	19570927
Plant Geography Investigation Team 603	PE	PE01374776	19780720
Plant Geography Investigation Team 603	PE	PE01374777	19780720
Qi-Guang Zhao 30441	PE	PE00866005	19390725
Qin-Er Yang 94138	PE	PE01869777	19990910
Qin-Er Yang 9606	PE	PE01869779	19960731
Qin-Er Yang et al. 180	PE	PE	19980829
Qin-Er Yang et al. 180	PE	PE	19980829
Qin-Er Yang et al. 180	PE	PE	19980829

Qin-Er Yang et al. 180	PE	PE	19980829
Qin-Er Yang et al. 180	PE	PE	19980829
Qin-Er Yang et al. 180	PE	PE	19980829
Qin-Er Yang et al. 180	PE	PE	19980829
Qin-Er Yang et al. 180	PE	PE	19980829
Qin-Er Yang et al. 452	PE	PE	19980921
Qin-Er Yang et al. 452	PE	PE	19980921
QinLin Team 10612	PE	PE	19580915
QinLin Team 10612	PE	PE	19580915
Qi-Wu Wang 63972	PE	PE00866024	19350600
Qi-Wu Wang 63972	PE	PE00866025	19350600
Qi-Wu Wang 64600	PE	PE00866022	19350700
Qi-Wu Wang 64600	PE	PE00866023	19350700
Qi-Wu Wang 67261	PE	PE00866016	19351000
Qi-Wu Wang 67261	PE	PE00866017	19351000
Qi-Wu Wang 68534	PE	PE00866020	19350800
Qi-Wu Wang 68534	PE	PE00866021	19350800
Qi-Wu Wang 68630	PE	PE00866026	19350800
Qi-Wu Wang 68630	PE	PE00866027	19350800
Qi-Wu Wang 68768	PE	PE00866018	19350800
Qi-Wu Wang 68768	PE	PE00866019	19350800
RC Ching 20994	PE	PE00866006	19390715
Ren-Chang Qin 23419	PE	PE00865998	19290929
Ren-Chang Qin 23419	PE	PE00865999	19290929
Ren-Chang Qin 24016	PE	PE00865996	19290823
Ren-Chang Qin 24016	PE	PE00865997	19290823
Ren-Chang Qin 24370	PE	PE00866000	19290906
Ren-Chang Qin 24370	PE	PE	19290906
RP Farges	PE	PE01899425	
Ru Long et al. 090807003	PE	PE01857430	20090807
Ru Long et al. 090807003	PE	PE01857431	20090807
Ru Long et al. 090807003	PE	PE01857432	20090807
Ru Long et al. 090807003	PE	PE01857433	20090807
Shen-E Liu 21278	PE	PE00865980	19461005
Sheng-Xiang Yu et al. 4603	PE	PE	20110730
Sheng-Xiang Yu et al. 4603	PE	PE	20110730
Sheng-Xiang Yu et al. 4875	PE	PE	20110803
Sheng-Xiang Yu et al. 4875	PE	PE	20110803
Shu-Zhen Gao 1212	PE	PE00865905	19550000
Su-Gong Wu 2138	PE	PE00865937	19590725
TaiBai Team 55242	PE	PE	19550712
TaiBai Team 55242	PE	PE	19550712
TH Tu 589	PE	PE00866140	19350813
Tibet Team 12215	PE	PE00864210	19830721

Tibet Team 12215	PE	PE00865918	19830721
Tibet Team 13052	PE	PE00864212	19830819
Tibet Team 13174	PE	PE00861989	19830821
Tibet Team 13174	PE	PE00861990	19830821
Tibet Team 2189	PE	PE00864202	19810706
Tibet Team 2189	PE	PE00864203	19810706
Tibet Team 2254	PE	PE00864201	19810709
Tibet Team 2254	PE	PE00864209	19810709
Tibet Team 2308	PE	PE00864204	19810710
Tibet Team 2308	PE	PE01982480	19810710
Tibet Team 2518	PE	PE00861991	19810709
Tibet Team 2518	PE	PE01982474	19810709
Tibet Team 8556	PE	PE00864205	19820725
Tibet Team 8556	PE	PE01982473	19820725
TN Liou & PC Tsoong 412	PE	PE00865899	19370829
TN Liou & PC Tsoong 412	PE	PE00865900	19370829
TN Liou & PC Tsoong 711	PE	PE00865898	19370908
TP Wang 1675	PE	PE00865901	19330801
TP Wang 1675	PE	PE00865902	19330801
TP Wang 1675	PE	PE00865903	19330801
TP Wang 1675	PE	PE00865904	19330801
Tsun-shen Ying 333	PE	PE	19870723
Tsun-shen Ying 333	PE	PE	19870723
Tsun-Shen Ying et al. 0333	PE	PE01536763	19870723
TT Yu 12079	PE	PE00866034	19370709
TT Yu 12079	PE	PE00866035	19370709
TT Yu 12079	PE	PE00866045	
TT Yu 12194	PE	PE00866032	19370716
TT Yu 12194	PE	PE00866033	19370716
TT Yu 12194	PE	PE00866054	
TT Yu 15397	PE	PE00866028	19370803
TT Yu 15397	PE	PE00866029	19370803
TT Yu 15397	PE	PE00866050	
TT Yu 16946	PE	PE00866010	19370724
TT Yu 16946	PE	PE00866011	19370724
TT Yu 16946	PE	PE00866012	19370724
TT Yu 16946	PE	PE00866047	
TT Yu 16946	PE	PE00866052	
TT Yu 22207	PE	PE00865984	19380806
TT Yu 22207	PE	PE00865985	19380806
TT Yu 22402	PE	PE00865986	19380818
TT Yu 22402	PE	PE00865987	19380818
TT Yu 22402	PE	PE00865989	19370818
TT Yu 22493	PE	PE00865990	19370826

TT Yu 22493	PE	PE00865991	19370826
TT Yu 22493	PE	PE00865992	19370826
TT Yu 22600	PE	PE00865993	19380831
TT Yu 22600	PE	PE00865994	19380831
TT Yu 22600	PE	PE00865995	19380831
TT Yu 22782	PE	PE00865982	19371008
TT Yu 22782	PE	PE00865983	19371008
TT Yu 22782	PE	PE00866015	19371008
TT Yu 3931	PE	PE00865939	19340831
TT Yu 3931	PE	PE00865957	19340831
TT Yu 6992	PE	PE00865946	19370711
TT Yu 6992	PE	PE00865947	19370711
TT Yu 6992	PE	PE00866046	
TT Yu 7226	PE	PE00865948	19370704
TT Yu 7226	PE	PE00865949	19370704
TT Yu 7603	PE	PE00865943	19370805
TT Yu 7603	PE	PE00865944	19370805
TT Yu 7603	PE	PE00865945	19370805
TT Yu 8894	PE	PE00866036	19370711
TT Yu 8894	PE	PE00866037	19370711
TT Yu 8894	PE	PE00866055	
TT Yu 8971	PE	PE00866038	19370714
TT Yu 8971	PE	PE00866039	19370714
TT Yu 8971	PE	PE00866048	
TT Yu 8971	PE	PE00866051	
TT Yu 9787	PE	PE00866030	19370823
TT Yu 9787	PE	PE00866031	19370823
TT Yu 9787	PE	PE00866049	
Unknown Collector	PE	PE01869774	19750908
Unknown Collector 0586	PE	PE00865922	19590609
Unknown Collector 0586	PE	PE00865923	19590609
Unknown Collector 10612	PE	PE01387860	19580915
Unknown Collector 10838	PE	PE00865936	19580717
Unknown Collector 11455	PE	PE00866057	19380828
Unknown Collector 13052	PE	PE00864211	19830819
Unknown Collector 135	PE	PE00865915	19730805
Unknown Collector 1568	PE	PE00865924	19590802
Unknown Collector 1568	PE	PE00865925	19590802
Unknown Collector 1853	PE	PE00865920	19580201
Unknown Collector 1853	PE	PE00865921	19580201
Unknown Collector 21124	PE	PE00866042	
Unknown Collector 34041	PE	PE01869783	19770813
Unknown Collector 34480	PE	PE00865886	19590708
Unknown Collector 34480	PE	PE00865887	19590708

Unknown Collector 35112	PE	PE00865885	19590826
Unknown Collector 3814	PE	PE00865932	19590711
Unknown Collector 3814	PE	PE00865938	19590711
Unknown Collector 3823	PE	PE00865933	19590712
Unknown Collector 4022	PE	PE00865934	19590719
Unknown Collector 5654	PE	PE00865913	
Unknown Collector 63836	PE	PE00865955	
Unknown Collector 822	PE	PE00865958	19230000
Unknown Collector 960381	PE	PE	19960712
Unknown Collector 960381	PE	PE	19960712
Unknown Collector 960383	PE	PE	19960712
Unknown Collector 960383	PE	PE	19960712
Unknown Collector 9796	PE	PE00865966	19600707
Unknown Collector s.n	PE	PE00866056	
Vegetation Research Team 12316	PE	PE00865935	19760719
Wei-Lie Chen et al. 8671	PE	PE00862149	19840728
Wen-Guang Hu & Zhu He 10426	PE	PE00865790	19510719
Wen-Guang Hu & Zhu He 10487	PE	PE00865791	19510724
Wen-Pei Fang 17597	PE	PE00865952	19410820
Wen-Pei Fang 2908	PE	PE00865940	19280813
Wen-Pei Fang 2908	PE	PE00866143	19280813
Wen-Pei Fang 3549	PE	PE00865942	19280925
Xiao-Hua Jin et al. DLJ-ET 1673	PE	PE02003642	20080708
Xiao-Hua Jin et al. SET-ET 45	PE	PE	20090812
Xiao-Hua Jin et al. ST2071	PE	PE01979365	20130805
Xiao-Hua Jin et al. ST2071	PE	PE01979366	20130805
Xiao-Hua Jin et al. YN-ET 1483	PE	PE	20090716
Xun-Lin Yu et al. 080026	PE	PE01869781	20080807
Xun-Lin Yu et al. 80026	PE	PE	20080807
Xun-Lin Yu et al. 80026	PE	PE	20080807
Y Tsiang 11510	PE	PE00866053	
Yao-Dong Chen et al. 2067	PE	PE00861987	19880930
Yao-Dong Chen et al. 2067	PE	PE00862148	19880930
You-Sheng Chen 4325	PE	PE	20020630
You-Sheng Chen 4325	PE	PE	20020630
Yunnan Tropical Biological Resources Comprehensive Inspection Team 2330	PE	PE00866044	
Zheng-Bo Feng et al. 4137	PE	PE01869780	20070727
Zheng-Yi Wu et al. 88	PE	PE01982475	19800825
Zhen-Shu Liu 822	PE	PE00865950	19340000
Zhi-Jian Yin et al. 1149	PE	PE01899016	20090713
Zhi-Jian Yin et al. 1265	PE	PE01898380	20090715
Zhi-Ying Zhang 16798	PE	PE00865907	

ZhongDian Team 1385	PE	PE00029360	19620826
ZhongDian Team 1385	PE	PE00865975	19620826
ZhongDian Team 1965	PE	PE00865976	19620909
ZhongDian Team 1965	PE	PE00865977	19620909
ZhongDian Team 850	PE	PE00865978	19620815
ZhongDian Team 850	PE	PE00865979	19620815
Zhong-Lun Wu 12277	PE	PE00865962	
Zhong-Lun Wu 12277	PE	PE00865964	
Zhu,Chen,Xu,Wang 1996	PE	PE01869776	19990910
Zi-Fu Song 39041	PE	PE00865953	19540000
Zuo-Bin Wang 15667	PE	PE00865892	19520708
Zuo-Bin Wang 16437	PE	PE00865894	19520903
BaiSha Team 452	SM	SM706501185	19790823
BaiSha Team 452	SM	SM	19790823
BaShan Collection Team 2286	PE	PE01851573	20080725
BaShan Collection Team 2499	PE	PE01851574	20080726
BaShan Collection Team 2499	PE	PE01851575	20080726
BeiChuan Team 382	SM	SM706501160	19780804
Chang-Lin Tang et al. 330	CDBI	CDBI0038769	19840821
Chang-Lin Tang et al. 330	CDBI	CDBI0038770	19840821
Chuan-Fen Wu 79-060	WCNU	WCNU0004750	19790708
Chuan-Fen Wu 79-060	WCNU	WCNU0004748	19790708
Da-Hua Du 589	SZ	SZ00179898	
Ding-Yi Peng 45999	CDBI	CDBI0038935	19820805
Ding-Yi Peng 45999	CDBI	CDBI0038936	19820805
Ding-Yi Peng 46552	CDBI	CDBI0038380	19820827
Ding-Yi Peng 46552	CDBI	CDBI0038934	19820827
FC Tai 5247	SZ	SZ00179876	19390927
Guang-Hui Yang 56455	SZ	SZ00179889	19570806
Guang-Hui Yang 56455	SZ	SZ00179874	19570806
Guang-Hui Yang 56633	SZ	SZ00179882	19570813
Guang-Hui Yang 56633	SZ	SZ00179888	19570813
Guang-Hui Yang 57412	SZ	SZ00179813	19570926
Guang-Hui Yang 57412	SZ	SZ00179875	19570926
Guang-Hui Yang 58765	SZ	SZ00179883	19580714
Guang-Hui Yang 58765	SZ	SZ00179894	19580714
Gui-Ling Qu 3231	SZ	SZ00179815	19360723
Gui-Ling Qu 3231	PE	PE00866145	19360000
Gui-Ling Qu 3231	PE	PE00866146	19250000
Gui-Ling Qu 3231	PE	PE00865960	19250000
Gui-Ling Qu 3524	SZ	SZ00179817	19360811
Guo-Feng Li 63836	SZ	SZ00179890	19570828
Guo-Feng Li 63836	SZ	SZ00179891	19570828
Guo-Feng Li 63877	SZ	SZ00179886	19570829

Guo-Feng Li 63877	SZ	SZ00179900	19570829
Guo-Mei Feng 21289	SZ	SZ00179893	19550901
Guo-Mei Feng 21631	SZ	SZ00180201	19551103
Hong-Fu Zhou & He-Yi Su 109914	SZ	SZ00179809	19640804
Hong-Fu Zhou & He-Yi Su 109914	SZ	SZ00179810	19640804
HongYa Group 1011	SM	SM706501182	19790816
HongYa Group 1011	SM	SM	19790816
LuShan Team 78-0819	SM	SM706501114	19780826
Ming-Yuan Fang 203	SZ	SZ00185050	19860705
MuXian 578	SM	SM706501202	19780805
MuXian 578	SM	SM	19780805
NanChang 1071	SM	SM706501200	19790708
NanChang 1071	SM	SM	19790708
NingZu 348	SM	SM706501201	19780714
NingZu 348	SM	SM	19780714
Parnassia leptophylla			
Pin-Yi Mao 00600	SZ	SZ00180202	19561103
Qing-Sheng Zhao & Ya-Bin Yang 7323	SZ	SZ00179974	19780711
Qing-Sheng Zhao & Ya-Bin Yang 7323	SZ	SZ00179976	19780711
Qing-Sheng Zhao et al. 6736	SZ	SZ00179824	19780723
Qing-Sheng Zhao et al. 6736	SZ	SZ00179970	19780723
Qing-Sheng Zhao et al. 6736	SZ	SZ00179972	19780723
Qing-Sheng Zhao et al. 6840	SZ	SZ00179831	19780728
Qing-Sheng Zhao et al. 6840	SZ	SZ00179969	19780728
Qing-Sheng Zhao et al. 6840	SZ	SZ00179971	19780728
Qing-Sheng Zhao et al. 6840	SZ	SZ00179973	19780728
Qing-Sheng Zhao et al. 7885	SZ	SZ00179827	19780731
Qing-Sheng Zhao et al. 7885	SZ	SZ00179821	19780731
Shan-Yong Chen et al. 4167	SZ	SZ00179878	19570820
Shi-Xue Zhang 79-237	WCNU	WCNU0004745	19790707
T.K.Wang 0605	SZ	SZ00179968	19380800
TC Lee 3342	SZ	SZ00179887	19400810
TC Lee 3514	SZ	SZ00179811	19400911
Third Group 555	WCNU	WCNU0004751	19790716
TianJin General Investigation 78-562	SM	SM706501113	19780801
Tong-Pei Yi 004	SZ	SZ00184779	19990919
Tong-Pei Yi 004	SZ	SZ00184786	19990919
Tong-Pei Yi 734	SZ	SZ00184870	19990920
Unknown Collector	SZ	SZ00179856	

Unknown Collector	SZ	SZ00179860	
Unknown Collector 001041	WCNU	WCNU0021102	19790803
Unknown Collector 0019	SZ	SZ00185049	19930703
Unknown Collector 002709	WCNU	WCNU0020223	19800815
Unknown Collector 002709	WCNU	WCNU0020224	19800815
Unknown Collector 002709	WCNU	WCNU0020225	19800815
Unknown Collector 00433	WCNU	WCNU0004756	19780806
Unknown Collector 00433	WCNU	WCNU0004755	19780806
Unknown Collector 11889	SM	SM706501204	19610910
Unknown Collector 120	SM	SM706501207	19780701
Unknown Collector 120	SM	SM	19780701
Unknown Collector 120	SM	SM	19780701
Unknown Collector 120	SM	SM	19780701
Unknown Collector 1568	SM	SM706501194	19590802
Unknown Collector 1853	SM	SM706501195	19590821
Unknown Collector 189	SZ	SZ00185051	
Unknown Collector 2	KUN	KUN0437280	19820903
Unknown Collector 20	SM	SM706501150	19780701
Unknown Collector 200470	SZ	SZ00377533	20040821
Unknown Collector 2138	SM	SM	
Unknown Collector 2167	WCNU	WCNU0004746	19770719
Unknown Collector 25044	SM	SM706501205	19600804
Unknown Collector 25044	SM	SM	19600804
Unknown Collector 25083	SM	SM706501203	19600807
Unknown Collector 25174	SM	SM706501206	19600814
Unknown Collector 2579	WCNU	WCNU	19730820
Unknown Collector 2579	WCNU	WCNU0004757	19730820
Unknown Collector 288	SM	SM706501196	19790718
Unknown Collector 288	SM	SM	19790718
Unknown Collector 3151	WCNU	WCNU	19800815
Unknown Collector 339	SM	SM	19780814
Unknown Collector 343	SM	SM706501199	19780702
Unknown Collector 343	SM	SM	19780702
Unknown Collector 343	SM	SM	19780702
Unknown Collector 414	SM	SM706501191	19790716
Unknown Collector 414	SM	SM	19790716
Unknown Collector 416	SM	SM706501192	19780809
Unknown Collector 416	SM	SM	19780809
Unknown Collector 4167	SM	SM706501188	19570820
Unknown Collector 4167	SM	SM	19570820
Unknown Collector 499	SM	SM706501193	19790629
Unknown Collector 499	SM	SM	19790629
Unknown Collector 644	SM	SM706501187	19780720
Unknown Collector 644	SM	SM	19780720

Unknown Collector 79077	SZ	SZ00179975	19790718
Unknown Collector 79-396	WCNU	WCNU0027555	19790718
Unknown Collector 79-396	WCNU	WCNU0027556	19790718
Unknown Collector 79-396	WCNU	WCNU0027557	19790718
Unknown Collector 99113	SZ	SZ00184752	19990731
Unknown Collector wl-3101	WCNU	WCNU00015877	20160905
Unknown Collector wl-3101	WCNU	WCNU00015879	20160905
Unknown Collector wl-3101	WCNU	WCNU00015876	20160905
Unknown Collector wl-3101	WCNU	WCNU00015880	20160905
Unknown Collector wl-3101	WCNU	WCNU00015881	20160905
Unknown Collector wl-3101	WCNU	WCNU00015882	20160905
WanYuan Team 534	SM	SM706501186	19780801
WanYuan Team 534	SM	SM	19780801
Wei-Kai Bao et al. 1399	CDBI	CDBI0040162	19930817
Wei-Kai Bao et al. 1399	CDBI	CDBI0040153	19930817
Wen-Guang Hu & Zhu He 10426	SZ	SZ00179828	19510719
Wen-Guang Hu & Zhu He 10426	SZ	SZ00179829	19510719
Wen-Guang Hu & Zhu He 10487	SZ	SZ00179822	19510724
Wen-Guang Hu & Zhu He 10487	SZ	SZ00179825	19510724
Wen-Guang Hu & Zhu He 11130	SZ	SZ00179826	19510906
Wen-Guang Hu 1212	SZ	SZ00179868	19390900
Wen-Guang Hu 13420	SZ	SZ00179862	19520909
Wen-Guang Hu 140	SZ	SZ00179880	19530817
Wen-Pei Fang & Wen-Guang Hu 51-533	SZ	SZ00179869	19540716
Wen-Pei Fang 17597	SZ	SZ00179877	19410820
Wen-Pei Fang 20844	SZ	SZ00179871	19520904
Wen-Pei Fang 51-848	SZ	SZ00179867	19540730
Wen-Pei Fang 561	KUN	KUN0437281	
Xing-Lin Jiang & Xiu-Shi Zhang 31757	SZ	SZ00179812	19520723
Xing-Lin Jiang & Xiu-Shi Zhang 31889	SZ	SZ00179873	19520802
Xing-Lin Jiang & Xiu-Shi Zhang 32684	SZ	SZ00179892	19520907
Xing-Lin Jiang 10832	SZ	SZ00179818	19580717
Xing-Lin Jiang 10838	SZ	SZ00179820	19580717
Xing-Lin Jiang 36411	SZ	SZ00179816	19530723
Xi-Tao Cai 53724	SZ	SZ00179830	19330817
Xi-Tao Cai 53860	SZ	SZ00249779	19330727
Xiu-Ying Hu 1343	SZ	SZ00179966	19390708
Xun-Sun Yu 06451	WCNU	WCNU0004747	19740705
YaAn Team 927	SM	SM706501183	19780813
YaAn Team 927	SM	SM706501184	19780813

Ya-Bin Yang 135	CDBI	CDBI0039720	19730805
Ya-Bin Yang 135	CDBI	CDBI0039586	19730805
Ya-Bin Yang 7323	SZ	SZ00179832	19780711
Ya-Bin Yang 7323	SZ	SZ00179977	19780711
Yi-Lin Chen 8519	CDBI	CDBI0038395	19850900
Ying Jiang 11510	SZ	SZ00179823	19330400
Yuan Han 599	SM	SM706501198	19780819
Yu-Hui Tao 51484	SZ	SZ00179881	19560815
Yu-Hui Tao 51484	SZ	SZ00179895	19560815
Yu-Hui Tao 51484	SZ	SZ00180081	19560815
Yu-Hui Tao 51604	SZ	SZ00179896	19560819
Yu-Hui Tao 51604	SZ	SZ00179897	19560819
Yu-Hui Tao 51604	SZ	SZ00179899	19560819
ZhaoJueJiCha Team 655	SM	SM706501197	19790630
ZhaoJueJiCha Team 655	SM	SM	19790630
ZhengHe Research Team 003709	WCNU	WCNU0020222	19800815
Zhen-Ju Zhao et al. 114897	SZ	SZ00180129	19810711
Zhen-Ju Zhao et al. 115239	SZ	SZ00180128	19810817
Zhen-Shu Liu 822	SZ	SZ00179814	
Zhong-Ming Tan & Shu-Hua Yu 123124	SZ	SZ00180143	19870810
Zhong-Ming Tan & Shu-Hua Yu 123124	SZ	SZ00180144	19870810
Zhong-Ming Tan & Shu-Hua Yu 123124	SZ	SZ00180145	19870810
Zhong-Ming Tan & Shu-Hua Yu 123125	SZ	SZ00180233	19870810
Zhong-Ming Tan & Shu-Hua Yu 123125	SZ	SZ00180234	19870810
Zhong-Ming Tan & Shu-Hua Yu 123125	SZ	SZ00180237	19870810
Zhong-Ming Tan & Shu-Hua Yu 123128	SZ	SZ00180140	19870810
Zhong-Ming Tan & Shu-Hua Yu 123128	SZ	SZ00180255	19870810
Zhong-Ming Tan & Shu-Hua Yu 123128	SZ	SZ00180256	19870810
Zhong-Ming Tan & Shu-Hua Yu 123129	SZ	SZ00180146	19870810
Zhong-Ming Tan & Shu-Hua Yu 123129	SZ	SZ00180152	19870810
Zhong-Ming Tan & Shu-Hua Yu 123129	SZ	SZ00180161	19870810

Zhong-Ming Tan & Shu-Hua Yu 123130	SZ	SZ00180147	19870810
Zhong-Ming Tan & Shu-Hua Yu 123130	SZ	SZ00180148	19870810
Zhong-Ming Tan & Shu-Hua Yu 123130	SZ	SZ00180151	19870810
Zhong-Ming Tan & Shu-Hua Yu 123141	SZ	SZ00180250	19870810
Zhong-Ming Tan & Shu-Hua Yu 123141	SZ	SZ00180251	19870810
Zhong-Ming Tan & Shu-Hua Yu 123141	SZ	SZ00180253	19870810
Zhong-Ming Tan & Shu-Hua Yu 123147	SZ	SZ00180248	19870810
Zhong-Ming Tan & Shu-Hua Yu 123147	SZ	SZ00180249	19870810
Zhong-Ming Tan & Shu-Hua Yu 123147	SZ	SZ00180252	19870810
Zhong-Ming Tan & Shu-Hua Yu 123199	SZ	SZ00180142	19870811
Zhong-Ming Tan & Shu-Hua Yu 123199	SZ	SZ00180149	19870811
Zhong-Ming Tan & Shu-Hua Yu 123199	SZ	SZ00180150	19870811
Zhong-Ming Tan & Shu-Hua Yu 123213	SZ	SZ00180125	19870811
Zhong-Ming Tan & Shu-Hua Yu 123213	SZ	SZ00180205	19870811
Zhong-Ming Tan & Shu-Hua Yu 123213	SZ	SZ00180206	19870811
Zhong-Ming Tan & Shu-Hua Yu 87010	SZ	SZ00180228	19870829
Zhong-Ming Tan & Shu-Hua Yu 87010	SZ	SZ00180229	19870829
Zhong-Ming Tan & Shu-Hua Yu 87010	SZ	SZ00180242	19870829
Zhong-Ming Tan & Shu-Hua Yu 87025	SZ	SZ00180137	19870829
Zhong-Ming Tan & Shu-Hua Yu 87025	SZ	SZ00180225	19870829
Zhong-Ming Tan & Shu-Hua Yu 87025	SZ	SZ00180227	19870829
Zhong-Ming Tan & Shu-Hua Yu 87031	SZ	SZ00180226	19870829

Zhong-Ming Tan & Shu-Hua Yu 87048	SZ	SZ00180138	19870831
Zhong-Ming Tan & Shu-Hua Yu 87048	SZ	SZ00180139	19870831
Zhong-Ming Tan & Shu-Hua Yu 87048	SZ	SZ00180214	19870831
Zhong-Ming Tan & Shu-Hua Yu 87052	SZ	SZ00180212	19870831
Zhong-Ming Tan & Shu-Hua Yu 87052	SZ	SZ00431950	19870831
Zhong-Ming Tan & Shu-Hua Yu 87052	SZ	SZ00180213	19870831
Zhong-Ming Tan & Shu-Hua Yu 87054	SZ	SZ00180210	19870831
Zhong-Ming Tan & Shu-Hua Yu 87054	SZ	SZ00180240	19870831
Zhong-Ming Tan & Shu-Hua Yu 87054	SZ	SZ00180241	19870831
Zhong-Ming Tan & Shu-Hua Yu 87055	SZ	SZ00180211	19870831
Zhong-Ming Tan & Shu-Hua Yu 87055	SZ	SZ00180243	19870831
Zi-Fu Song	SZ	SZ00179863	19540000
Zi-Fu Song 39041	SZ	SZ00179819	19540714
Zi-Fu Song 414068	SZ	SZ00179967	19540000
